# Omeprazole activation of CD4+ and CD8+ T-cells through off-target covalent modification of cellular proteins

**DOI:** 10.1093/toxsci/kfag046

**Published:** 2026-04-27

**Authors:** Sophie Grice, Sa’d Albashtawy, Georgia Wells, Luisa Hering, Kareena Adair, Joscelyn Sarsby, Philip Brownridge, Megan Ford, Rachel Lloyd, Lucy Hampson, Annette Wagner, Yonghu Sun, Hong Liu, Sean Hammond, Xiaoli Meng, Furen Zhang, Dean Naisbitt

**Affiliations:** Department of Pharmacology and Therapeutics, University of Liverpool, Liverpool L69 3GE, United Kingdom; Department of Pharmacology and Therapeutics, University of Liverpool, Liverpool L69 3GE, United Kingdom; Department of Pharmacology and Therapeutics, University of Liverpool, Liverpool L69 3GE, United Kingdom; Department of Pharmacology and Therapeutics, University of Liverpool, Liverpool L69 3GE, United Kingdom; Centre for Proteome Research, University of Liverpool, Liverpool L69 7ZB, United Kingdom; Centre for Proteome Research, University of Liverpool, Liverpool L69 7ZB, United Kingdom; Centre for Proteome Research, University of Liverpool, Liverpool L69 7ZB, United Kingdom; Department of Pharmacology and Therapeutics, University of Liverpool, Liverpool L69 3GE, United Kingdom; Department of Pharmacology and Therapeutics, University of Liverpool, Liverpool L69 3GE, United Kingdom; Department of Pharmacology and Therapeutics, University of Liverpool, Liverpool L69 3GE, United Kingdom; Department of Adult Allergy, Guy’s and St Thomas’ Hospital, London SE1 9RT, United Kingdom; Shandong Provincial Institute of Dermatology and Venereology, Shandong Academy of Medical Sciences, Jinan, Shandong 250022, China; Shandong Provincial Institute of Dermatology and Venereology, Shandong Academy of Medical Sciences, Jinan, Shandong 250022, China; Department of Pharmacology and Therapeutics, University of Liverpool, Liverpool L69 3GE, United Kingdom; ApconiX, Alderley Edge SK10 4TG, United Kingdom; Department of Pharmacology and Therapeutics, University of Liverpool, Liverpool L69 3GE, United Kingdom; Shandong Provincial Institute of Dermatology and Venereology, Shandong Academy of Medical Sciences, Jinan, Shandong 250022, China; Department of Pharmacology and Therapeutics, University of Liverpool, Liverpool L69 3GE, United Kingdom

**Keywords:** targeted covalent inhibitor drugs, omeprazole, T-lymphocytes, hapten, crossreactivity, allergy

## Abstract

Targeted covalent inhibition of protein function is increasingly used as a therapeutic mode of action; however, there is a need to characterize off-target binding interactions and to understand whether this represents an immunological risk. Given that the proton-pump inhibitor omeprazole exerts its mechanism of action through covalent inhibition, it serves as an ideal model to investigate the relationship between off-target protein binding and T-cell activation. Binding of omeprazole, omeprazole metabolites, and alternative proton-pump inhibitors to antigen-presenting cells and GST-pi was characterized by mass spectrometry. Omeprazole-responsive clones were generated and assessed in terms of cytokine secretion, pathways of T-cell activation, and crossreactivity with omeprazole metabolites, alternative proton-pump inhibitors, and unrelated drugs. Omeprazole stimulated CD4+ and CD8+ T-cell clones to proliferate and secrete cytokines and cytolytic molecules. HLA-restricted T-cell activation was dependent on processing of omeprazole protein adducts by antigen-presenting cells. Omeprazole-modified CYS-containing peptides derived from 36 off-target proteins were detected within antigen-presenting cells. Omeprazole metabolites and alternative protein pump inhibitors that form protein adducts also activated omeprazole-responsive T-cells. In conclusion, T-cells were activated with omeprazole via a hapten mechanism and exhibited considerable promiscuity to metabolites and structurally related drugs of the same pharmacological class. Similar off-target binding interactions may be a relevant concern for the increasing number of covalent inhibitor drugs receiving regulatory approval.

T-cells from patients with delayed-type drug hypersensitivity reactions are stimulated through a specific interaction of the culprit drug with MHC proteins and MHC binding peptide. With the exception of abacavir (Illing et al. 2012; Ostrov et al. 2012; Thomson et al. 2020), drugs are assumed to interact with the MHC binding peptide on the outer surface providing additional bonding energy with T-cell receptors and hence imparting a degree of triggering selectivity (Adair et al. 2021; Pichler 2022). A plethora of studies have explored the nature of the binding interaction of drugs with MHC-associated peptides. Hapten theory states that chemically reactive drugs, such as penicillins, bind covalently to proteins and that processed drug-modified peptides interact with MHC proteins to initiate a T-cell response (Padovan et al. 1997; Meng et al. 2017; Azoury et al. 2018; Waddington et al. 2020). Other drugs are not directly protein reactive and do not form covalently modified MHC-associated drug-peptide conjugates. Studies by Pichler and colleagues demonstrated that such drugs interact directly with MHC-associated peptides through the formation of noncovalent bonds (Schnyder et al. 1997; Zanni et al. 1997, 1998), which explains why they can be used in in vitro diagnostic testing (e.g. lymphocyte transformation test, ELISpot) and functional T-cell studies to define the nature of the immune response induced in patients presenting with hypersensitivity (Britschgi et al. 2001; Whitaker et al. 2011; Zhao et al. 2019; Villani et al. 2021).

Proton pump inhibitors such as omeprazole are a class of drugs with widespread use for the treatment of peptic ulcer disease and gastroesophageal reflux disease (Fig. 1). Indeed, some 73 million courses were prescribed in the United Kingdom during 2022-2023 to around ∼15% of the population (Abrahami et al. 2020; Jenkins and Modolell 2023). This class of drugs exerts their mechanism of action through a covalent binding mechanism of action; under acidic conditions, omeprazole is transformed into a sulfenamide, which binds covalently to and inhibits H^+^/K^+^ ATPases (proton pumps) expressed by parietal cells. A principal concern of covalent binding drugs is that the covalent adduction of proteins can lead to the generation of drug-specific antigenic determinants and ultimately immunogenicity-related hypersensitivity risks as supported by >90 yr of hapten research and has been a key driver in the general avoidance of direct or bioactivation-related chemical reactivity in drug design for several decades (Park et al. 2011). The safety profile of proton pump inhibitors is generally acceptable; however, there remains a low risk of immune-mediated adverse events. Omeprazole is known to cause immediate and delayed-type hypersensitivity reactions (Kepil Ozdemir et al. 2016; Lin et al. 2018; Kepil Ozdemir and Bavbek 2020; Mori et al. 2020; Bavbek et al. 2024; Pinyopornpanish et al. 2024). A recent study reported on the clinical features of 69 cases of delayed onset reactions induced by proton pump inhibitors (Lin et al. 2018). Omeprazole and esomeprazole accounted for 48 of the cases with clinical presentations including maculopapular exanthema, Stevens Johnson syndrome, acute generalized exanthematous pustulosis, and drug rash with eosinophilia and systemic symptoms (DRESS).

**Fig. 1.**
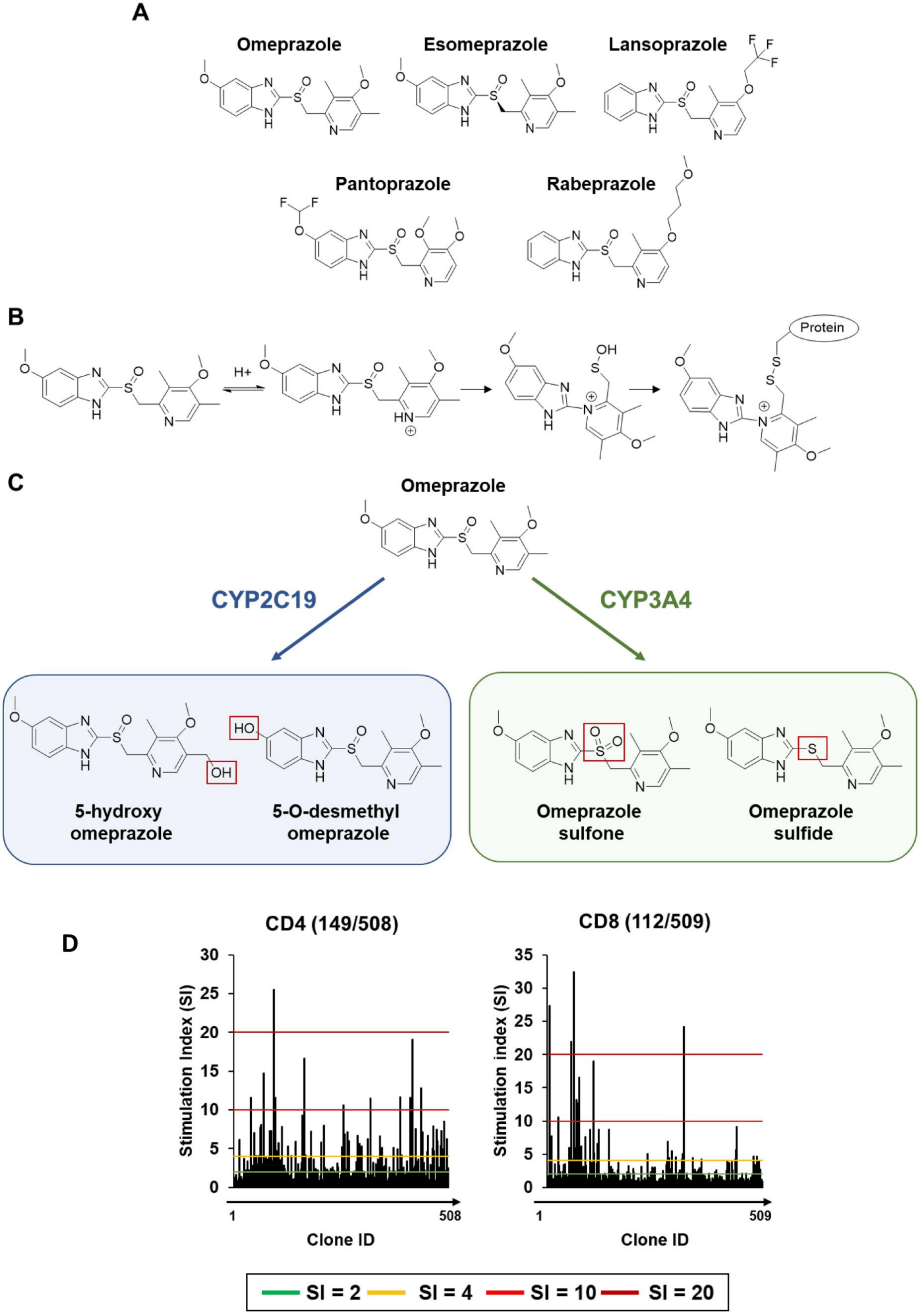
**Structure of omeprazole, proton pump inhibitors, and generation of omeprazole-responsive CD4+ and CD8+ T-cell clones. (A)** Chemical structure of proton pump inhibitors. **(B)** Depiction of the mechanism of action of omeprazole through formation of a sulfonamide intermediate under acidic conditions enabling binding to the proton pump. **(C)** Omeprazole metabolism by CYP2C19 and CYP3A4. **(D)** T-cells were expanded from single precursors and tested for omeprazole proliferative responses using irradiated autologous EBV-transformed B-cells as antigen-presenting cells. [^3^H]thymidine was added for the final 16 h of the culture period and proliferative responses measured using scintillation counting. Stimulation index (SI) was calculated by dividing the average CPM of drug-treated conditions by the average CPM of the medium control; clones displaying an SI of above 1.5 were expanded and analyzed in dose-response studies. SI of 2 (green), 4 (yellow), 10 (red), and 20 (dark red) are displayed with lines on the figure.

Peripheral blood mononuclear cells (PBMC) from patients with delayed-type omeprazole hypersensitivity reactions are stimulated to proliferate and secrete effector molecules in vitro in the presence of the drug (Lin et al. 2018; Pinyopornpanish et al. 2024); however, mechanistic studies exploring the relationship between off-target protein binding and T-cell activation have not been performed. Herein, a panel of CD4+ and CD8+ T-cell clones was used alongside omeprazole, omeprazole metabolites, and additional proton pump inhibitors to show that off-target covalent binding is critical for the formation of crossreactive antigenic determinants that activate patient T-cells.

## Materials and methods

### Cell culture medium

PBMC and T-cells were cultured in R9 medium made by supplementing RPMI 1640 (Sigma Aldrich, St. Louis, MO, United States) with heat inactivated human blood type AB serum (5%; Innovative Research, Novi, MI, United States), L-glutamine (2 mM; Sigma Aldrich), HEPES (4-(2-hydroxyethyl)-1-piperazineethanesulfonic acid) (25 mM; Sigma Aldrich), transferrin (25 μg/ml; Sigma Aldrich), and penicillin-streptomycin solution (penicillin 100 IU/ml; streptomycin 100 μg/ml; Sigma Aldrich). T-cell clones were maintained in R9 enriched with human recombinant IL-2 (100 IU/ml; Peprotech, London, United Kingdom). Immortalized Epstein Barr virus (EBV)-transformed B-cell lines were generated by transformation of PBMC with supernatant from the EBV-producing cell line B95.8. EBV-transformed B-cell lines were maintained in F1 medium made by supplementing RPMI 1640 with fetal bovine serum (10%; Invitrogen, Waltham, MA, United States), L-glutamine (2 mM), HEPES (25 mM), and penicillin-streptomycin solution (penicillin 100 IU/ml; streptomycin 100 μg/ml) with the addition of cyclosporin A (1 μg/ml) for 3 wk followed by F1 medium alone thereafter. All compounds were cultured with EBV-transformed B-cells to determine dose ranges that were not associated with inhibition of proliferation or loss of cell viability prior to conducting T-cell assays.

### Characterization of drug(metabolite) glutathione S-transferase Pi (GSTP) adducts by mass spectrometry

GSTP has been used as a model protein to explore the binding interaction of thiol-reactive drugs, due to the presence of a highly reactive cysteine residue (Jenkins et al. 2008; Callan et al. 2009; Jenkinson et al. 2009; Meng et al. 2014; Yip et al. 2017). To explore the reactivity of omeprazole, omeprazole metabolites, and additional protein pump inhibitors, compounds (100 µM) were incubated with GSTP purified using HIS-Select Nickel Affinity Gel (Sigma-Aldrich, St. Louis, MO, United States) for 1-24 h. Iodoacetamide was added to alkylate free cysteine. Following protein quantification, trypsin (2 µg) (Promega, Madison, WI, United States) was added to 200 µg protein for 16 h and the resulting digests were further cleaned up using C18 ZipTips (Millipore, Bedford, MA, United States). Samples were reconstituted in 2% ACN, 0.1% FA (v/v), and analyzed by using a Triple TOF 6600 mass spectrometer (Sciex) coupled with an Eksigent NanoLC Ultra HPLC system. Samples were injected onto a nanoACQUITY UPLC Symmetry C18 Trap Column (P/N Waters, MA, United States) and washed for 10 min at 2 µl/min with 0.1% FA. A gradient from 1.6% ACN/0.1% FA to 95% ACN/0.1% FA was applied over 95 min at a flow rate of 300 nl/min through a Peptide BEH C18 nanoACQUITY Column (Waters, MA, United States). MS was operated as described in previous methods (Meng et al. 2014, 2016).

### Identification of omeprazole-modified antigen-presenting cell proteins by mass spectrometry

EBV-transformed B-cells were treated with omeprazole (100 µM) for 24 h in F1 cell culture medium. Cells were pelleted and lysed using lysis buffer (7.0 M urea, 2.0 M thiourea, 4% CHAPS, 40 mM Tris base). Purified cell lysates were digested using trypsin and the resulting tryptic peptides were further cleaned up using C18 ZipTips (Millipore, Bedford, MA, United States). Samples were reconstituted in 23% ACN, 0.1% FA (v/v) and 1 µl of sample was analyzed using an ultra-high-pressure nano-flow chromatography system (nanoElute, BrukerDaltonic) coupled to a TIMS quadrupole time-of-flight mass spectrometer (timsTOF SCP, Bruker Daltonics, Bremen, Germany). The sample was loaded onto the trapping column (Thermo Scientific, PepMap100, C18, 300 μm×5 mm) using 1 μl pick up with 4× injection volume plus 2 μl. The sample was then resolved on the analytical column (PepSep, 25 Series 25 cm×150 μm×1.5 μm column) at 20°C using a gradient of 98% A (0.1% formic acid)/2% B (acetonitrile/0.1% formic acid) increasing to 65% A/35% B over 30 min followed by a hold at 95% B for 12.5 min, at a flow rate of 0.5 μl/min.MS 1 scans were acquired from 100 to 1,700 m/z and 1/K0 = 1.3 V-s/cm^2^ to 0.7 V-s/cm^2^ in DDA-PASEF mode. For MS2 precursor ion isolation, 10 PASEF ramps were acquired with an accumulation and ramp times of 166 ms with a total cycle time of 1.89 s. Precursor ions with a charge minimum of 0 and maximum of 5, above the minimum intensity threshold of 500 were isolated with 2 Th at < 700 m/z or 3 Th >800 m/z and resequenced until a target intensity of 20,000, with a dynamic exclusion of 40 s. The collision energy was lowered as a function of increasing ion mobility from 59 eV at 1/K0 = 1.6 V-s/cm^2^ to 20 eV at 1/K0 = 0.6 V-s/cm^2^. Quality control experiments were conducted using HELA to identify protein and peptides to ensure sample quality.

LC-MS/MS data were searched against the reviewed human proteome (UniProt/SwissProt 2020), using PEAKS Studio 12 (Bioinformatics Solution Inc). Data were refined using default parameters, and PEAKS DB searches performed with the following parameters: Parent Mass Error Tolerance 10 ppm, Fragment Mass Error Tolerance 0.1 Da, no enzymatic restriction, Variable modifications-methionine oxidation (+15.99), asparagine and glutamine deamidation (+0.98), omeprazole modification of cysteine (+327.11). The maximum number of variable posttranslational modifications per peptide was 3. False discovery rate (FDR) was estimated with decoy-fusion. MS/MS spectra were also manually validated to confirm omeprazole modification.

### Generation of omeprazole-responsive CD4+ and CD8+ T-cell clones

T-cell clones were generated from a patient that developed atypical DRESS after being administered omeprazole. Clinical details of the reaction and results of diagnostic testing are available in Grice et al. (2024). Patient PBMC (1×10^6^) were incubated with omeprazole (12.5-50 μM) in R9 medium for 2 wk and supplemented with IL-2 (200 IU/ml) on day 6 and 9 to maintain proliferation. On day 14, PBMC were positively selected into CD4+ and CD8+ T-cell subsets using anti-CD4+ and anti-CD8+ microbeads (Miltenyi Biotec, Bisley, United Kingdom). T-cell lines were cloned by limiting dilution as described previously (Naisbitt et al. 2003). For specificity testing, T-cell clones (5×10^4^) were cultured with irradiated autologous EBV-transformed B-cells (1×10^4^) and R9 medium as the negative control or omeprazole (25 µM) for 48 h. After incubation, [^3^H]-thymidine was added, and proliferation was measured by scintillation counting. T-cell clones with a stimulation index (SI) ≥ 2 were considered omeprazole-responsive and expanded for phenotypic and mechanistic characterization.

### Phenotyping of omeprazole-responsive T-cell clones

T-cell clones (5×10^4^) were incubated with anti-CD8 PE (clone; RPA-T8, BD Biosciences, Oxford, United Kingdom) (1:100) and anti-CD4 APC (clone; RPA-T4, BD Biosciences, Oxford, United Kingdom) (1:33) antibodies, followed by washing and analysis using a BD FACS Canto II flow cytometer.

### Evaluation of cytokine and cytolytic molecule secretion from omeprazole-responsive T-cell clones

T-cell clones (5×10^4^) were cultured with drug, R9 medium (negative control), or phytohemagglutinin (PHA) (10 µg/ml) and irradiated autologous EBV-transformed B-cell lines (1×10^4^). ELISpot was performed according to manufacturer’s instructions (Mabtech, Nacka Strand, Sweden) to assess cytokine and cytolytic molecule secretion (IFN-γ, IL-5, IL-13, IL-22, IL-17, perforin, granzyme B). Cytokine and cytolytic molecule secretion was also quantified from culture supernatant (25 μl) using bead-based immunoassays according to the manufacturer’s instructions (LEGENDplex, BioLegend Custom Human 11-plex panel; granzyme B, IFN-γ, IL-10, IL-13, IL-22, IL-5, IL-6, perforin, sFasL, IL-17A, TNF-α). To assess IFN-γ, IL-13 and granzyme B expression intracellular cytokine staining was conducted utilizing flow cytometry. Cells were fixed and permeabilized before the addition of antibody cocktail containing anti-CD3 BV510 (clone; UCHT1, BioLegend, United Kingdom) (1:50), CD45RO PerCPCy5.5 (clone; UCHT1 BioLegend, United Kingdom) (1:50), CD4 FITC (clone; RPA-T4, BD Biosciences, Oxford, United Kingdom) (1:30), granzyme B PE-Cy7 (clone; QA16A02, BioLegend, United Kingdom) (1:100), IFN-γ APC (clone; 4S.B3, Thermo Fisher Scientific, United Kingdom) (1:200), IL-13 PE (clone; 85BRD, Thermo Fisher Scientific, United Kingdom) (1:25), and CD8 BV421 (clone; RPA-T8, BioLegend, United Kingdom) (1:100) antibodies. PMA (Sigma-Aldrich, Dorset, United Kingdom) (50 ng/ml) and ionomycin (Thermo Fisher Scientific, United Kingdom) (500 ng/ml) were used as the positive control. Cells were washed and analyzed on a BD FACS Canto II flow cytometer.

### Mechanism of drug presentation to omeprazole-responsive T-cell clones

Anti-HLA class I (BioLegend, United Kingdom), anti-HLA class II, and anti-HLA DR blocking antibodies (BD Biosciences) were added to proliferation assays to assess the involvement of drug-MHC binding in the activation of CD4+ and CD8+ T-cell clones. Incubations without EBV-transformed B-cell lines were conducted to assess self-presentation. The role of antigen processing in drug presentation to T-cell clones was determined by chemical fixation of autologous EBV-transformed B-cell with glutaraldehyde. Antigen-presenting cell protein processing is inhibited by fixation and as such only drugs which directly interact with surface MHC peptide complexes are presented to T-cells (Schnyder et al. 1997; Zanni et al. 1998). Fixation with glutaraldehyde does not prevent the presentation of preprocessed peptide antigens (Zanni et al. 1998); thus, glutaraldehyde does not alter the confirmation of HLA to prevent T-cell activation. Fixation may alter the activation of co-stimulatory pathways, but such pathways are not required for the activation of cloned memory T-cells. To evaluate the role of omeprazole protein binding in the activation of T-cells, omeprazole, proton pump inhibitors, and omeprazole metabolites were cultured with autologous EBV-transformed B-cells (2 × 10^6^) for 0.5-24 h. Repeated washing steps were conducted to remove unbound drug(metabolite) before incubation with T-cell clones. Finally, omeprazole antigen-presenting cell lysates were prepared and used as a source of drug antigen to activate clones. Briefly, EBV-transformed B cells (2×10^6^/ml) were incubated with omeprazole in R9 media (37°C, 5% CO_2_) for 16 h. Cells were then irradiated for 30 min to inhibit any proliferative potential and washed ×3 (1,500 RPM for 5 min) in R9 media. After the final wash, cells were resuspended in 1 ml R9 media and subjected to 5 min of ultrasonic sonication to lyse the cells and generate drug-protein lysates. Cell counts before sonication were used to resuspend cells to an appropriate dilution and “cell ratios” of drug-conjugates were added to each well. An absence of viable EBV-transformed B-cells was confirmed by microscopic evaluation with trypan blue dye exclusion.

### Statistical analysis

A 1-way ANOVA Dunnett’s multiple comparison test was carried for normally distributed data. If the data set was not normally distributed, a 1-way ANOVA Kruskal–Wallis Dunn’s multiple comparison test was carried out.

## Results

### Phenotypic and functional characterization of omeprazole-responsive T-cell clones

**Fig. 2.**
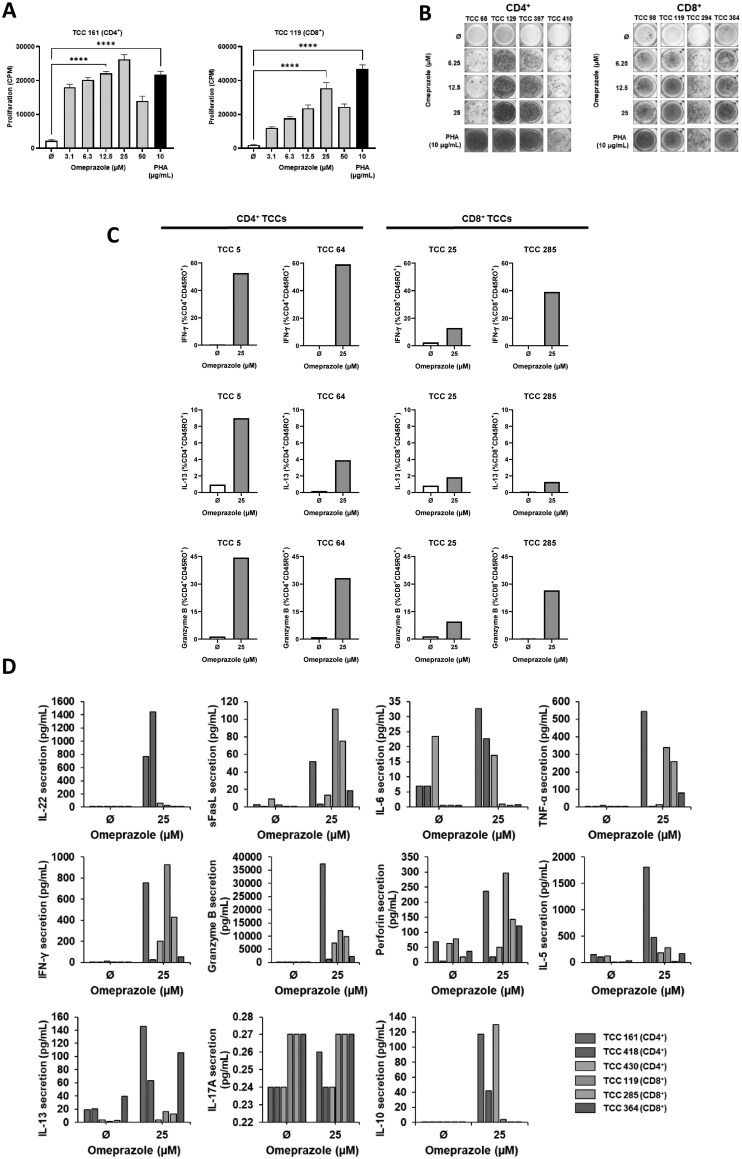
**Cytokine secretion from omeprazole-responsive CD4+ and CD8+ T-cell clones. (A)** CD4+ (*n* = 45) and CD8+ (*n* = 22) T-cell clones (5×10^4^) were cultured with irradiated autologous EBV-transformed B-cell lines as APCs (1×10^4^) and omeprazole. Proliferation was measured after 48 h via [^3^H]-thymidine incorporation. Data is presented as CPM ± SD. One representative CD4+ and CD8+ T-cell clone is presented. Statistical significance was determined using Dunnett’s multiple comparison 1-way ANOVA test (*P < 0.05, **P < 0.01, ***P < 0.001, ****P < 0.0001). **(B)** IFN-γ secretion was assessed from CD4+ (*n* = 4) and CD8+ (*n* = 4) T-cell clones using ELISpot assay **(C)** Flow cytometry was utilized to assess intracellular expression of IFN-γ, IL-13, and granzyme B from CD4+ (*n* = 2) and CD8+ (*n* = 2) T-cell clones**. (D)** Supernatant from 48 h incubations containing CD4+ (*n* = 3) and CD8+ (*n* = 3) T-cell clones, irradiated autologous EBV-transformed B-cells and omeprazole was utilized in a LEGENDplex assay to quantify secretion of IL-22, sFasL, IL-6, TNF-α, IFN-γ, granzyme B, perforin, IL-5, IL-13, IL-17A, and IL-10.

T-cell cloning via serial dilution was conducted with omeprazole-treated PBMC. After testing 1,017 single-cell cultures for specificity, 261 omeprazole-responsive T-cell clones were identified (Fig. 1D). Phenotyping was performed on 68 of the drug-responsive T-cell clones after expansion and second testing. Expression of CD4+ or CD8+ receptors on was found on 45 and 22 T-cell clones, respectively. The final clone expressed high levels of both CD4 and CD8 however it was not successfully expanded and characterized. CD4+ and CD8+ T-cell clones exhibited a significant dose-dependent proliferative response and IFN-γ secretion in response to omeprazole (3.1-50 µM) (Fig. 2A and B). Intracellular cytokine staining analysis confirmed T-cell clones as CD45RO+ memory cells and revealed expression of IFN-γ, granzyme B, and IL-13 from both CD4+ and CD8+ T-cell clones (Fig. 2C). ELISpot revealed dose-dependent secretion of IL-22 in 2/3 and IL-5, IL-13, granzyme B, and perforin in 3/3 CD4+ T-cell clones (Fig. S1A). Additionally, ELISpot revealed dose-dependent secretion of IL-5 in 1/3, IL-13 and perforin in 2/3, and granzyme B in 3/3 CD8+ T-cell clones (Fig. S1B). From the LEGENDplex assay secretion of IL-5, IL-13, IFN-γ, TNF-α, perforin, granzyme, and sFasL was detected from omeprazole-treated CD4+ and CD8+ T-cell clones, whereas secretion of IL-10, IL-6, and IL-22 was observed with CD4+ clones only (Fig. 2D).

Activation of CD4+ and CD8+ T-cell clones with omeprazole was HLA class II and I restricted, respectively. CD4+ clones displayed a reduction in proliferation with the addition of anti-MHC class II and anti-DR antibodies, whereas CD8+ clones displayed a reduction in proliferation with the addition of anti-MHC class I antibodies (Fig. S2).

### CD4+ and CD8+ omeprazole-responsive T-cell clones are cross-reactive with other proton pump inhibitors

**Fig. 3.**
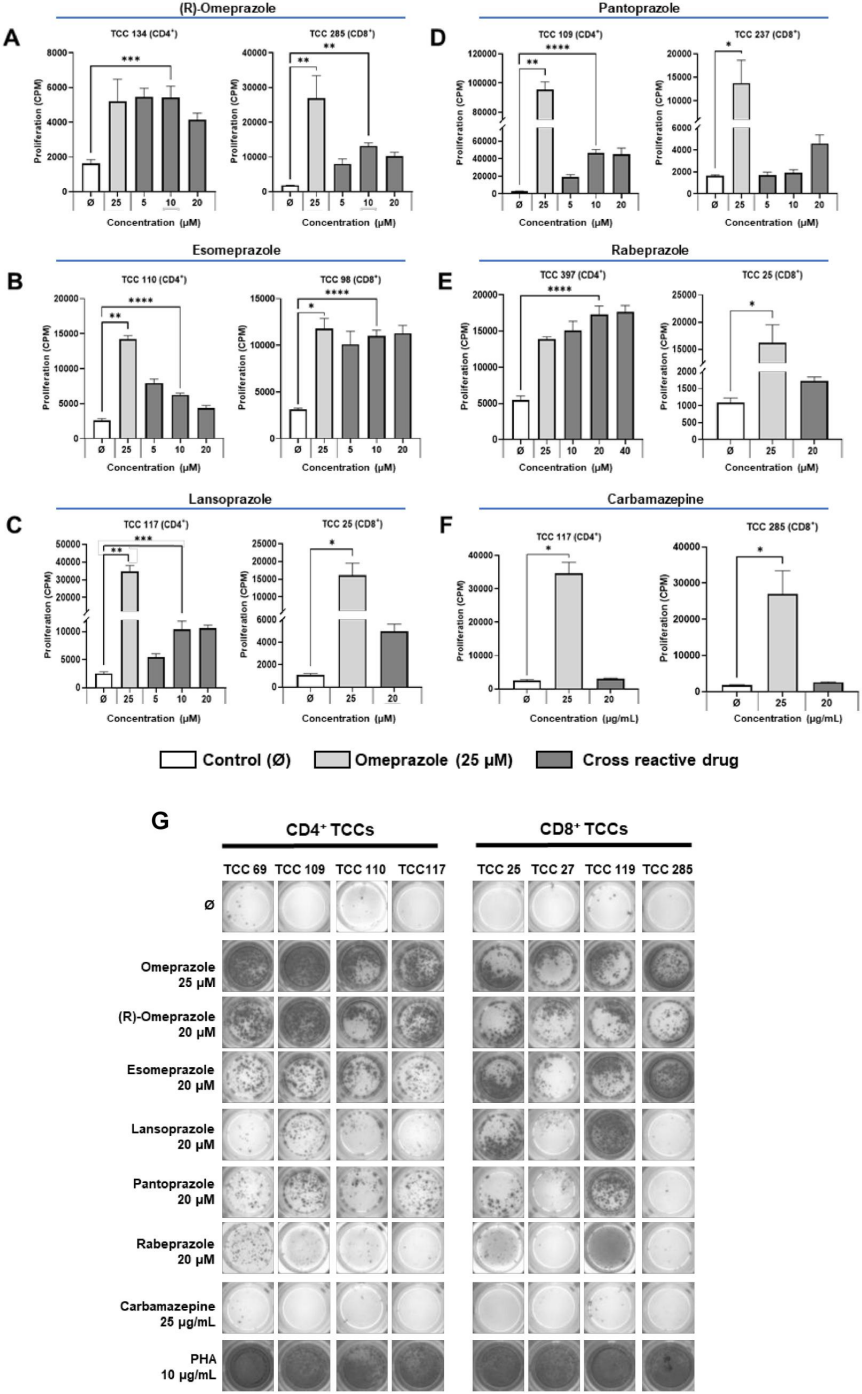
**CD4+ and CD8+ omeprazole-responsive T-cell clones display additional reactivity towards (R) and (S) enantiomers of omeprazole and other proton pump inhibitors**. CD4+ (*n* = 10) and CD8+ (*n* = 9) T-cell clones (5×10^4^) were cultured with irradiated autologous EBV-transformed B-cells (1×10^4^) and either **(A)** R-omeprazole, **(B)** S-omeprazole (esomeprazole), **(C)** lansoprazole, **(D)** pantoprazole, **(E)** rabeprazole, or **(F)** carbamazepine. Proliferation was assessed after 48 h via [^3^H]-thymidine incorporation and data are presented as CPM ± SD. One representative CD4+ and CD8+ T-cell clone is presented for each compound. Statistical significance was determined using Dunnett’s multiple comparison 1-way ANOVA test for parametric data and using Dunn’s multiple comparison Kruskal-Wallis 1-way ANOVA test for nonparametric data (*P < 0.05, **P < 0.01, ***P < 0.001, ****P < 0.0001). **(G)** CD4+ (*n* = 12) and CD8+ (*n* = 9) T-cell clones (5×10^4^) were cultured with irradiated autologous EBV-transformed B-cells (1×10^4^) and the test compounds, and IFN-γ secretion was assessed using ELISpot. Four representative CD4+ and CD8+ T-cell clones are presented.

Assessment of crossreactivity of omeprazole T-cell clones with single enantiomeric forms of omeprazole, proton pump inhibitors (Fig. 1), H2 antagonists famotidine and nizatidine, and an unrelated compound carbamazepine was conducted using [^3^H]-thymidine proliferation and ELIspot IFN-γ secretion as readouts. The majority of CD4+ and CD8+ T-cell clones proliferated and secreted IFN-γ in response to omeprazole (therapeutic form containing both enantiomeric forms), pure (R)-omeprazole, and esomeprazole (pure (S)-omeprazole). 25-70% of the clones tested were also stimulated to proliferate and/or secrete IFN-γ with other proton pump inhibitors (lansoprazole, pantoprazole, and rabeprazole) (Fig. 3, Table 1). In contrast, clones were not activated with H2 antagonists famotidine and nizatidine (Fig. S3), or carbamazepine (Fig. 3).

**Table 1.** Summary of crossreactivity of omeprazole-responsive CD4+ and CD8+ clones with the (R) enantiomer of omeprazole, structurally related proton pump inhibitors, omeprazole metabolites, and carbamazepine assessed using [^3^H]-thymidine proliferation and IFN-γ ELISpot readouts.

Readout	Phenotype	Omeprazole	Esomeprazole	Lansoprazole	Pantoprazole	Rabeprazole	(R)-Omeprazole	5-Hydroxy omeprazole	5-O-desmethyl omeprazole	Omeprazole sulfide	Omeprazole sulfone	Carbamazepine
**IFN-γ ELISpot *n* = 21**	CD4+ *n* = 12	12/12	11/12	6/12	6/12	6/12	12/12	11/12	9/12	3/12	12/12	0/12
	CD8+ *n* = 9	9/9	9/9	2/9	3/9	2/9	9/9	9/9	8/9	1/9	6/9	0/9
**3-H thymidine *n* = 19**	CD4+ *n* = 10	10/10	10/10	4/10	7/10	3/10	10/10	10/10	7/10	2/10	10/10	0/10
	CD8+ *n* = 9	9/9	9/9	2/9	4/9	1/9	9/9	9/9	8/9	0/9	7/9	0/9

### CD4+ and CD8+ omeprazole-responsive T-cell clones are cross-reactive with omeprazole metabolites

**Fig. 4.**
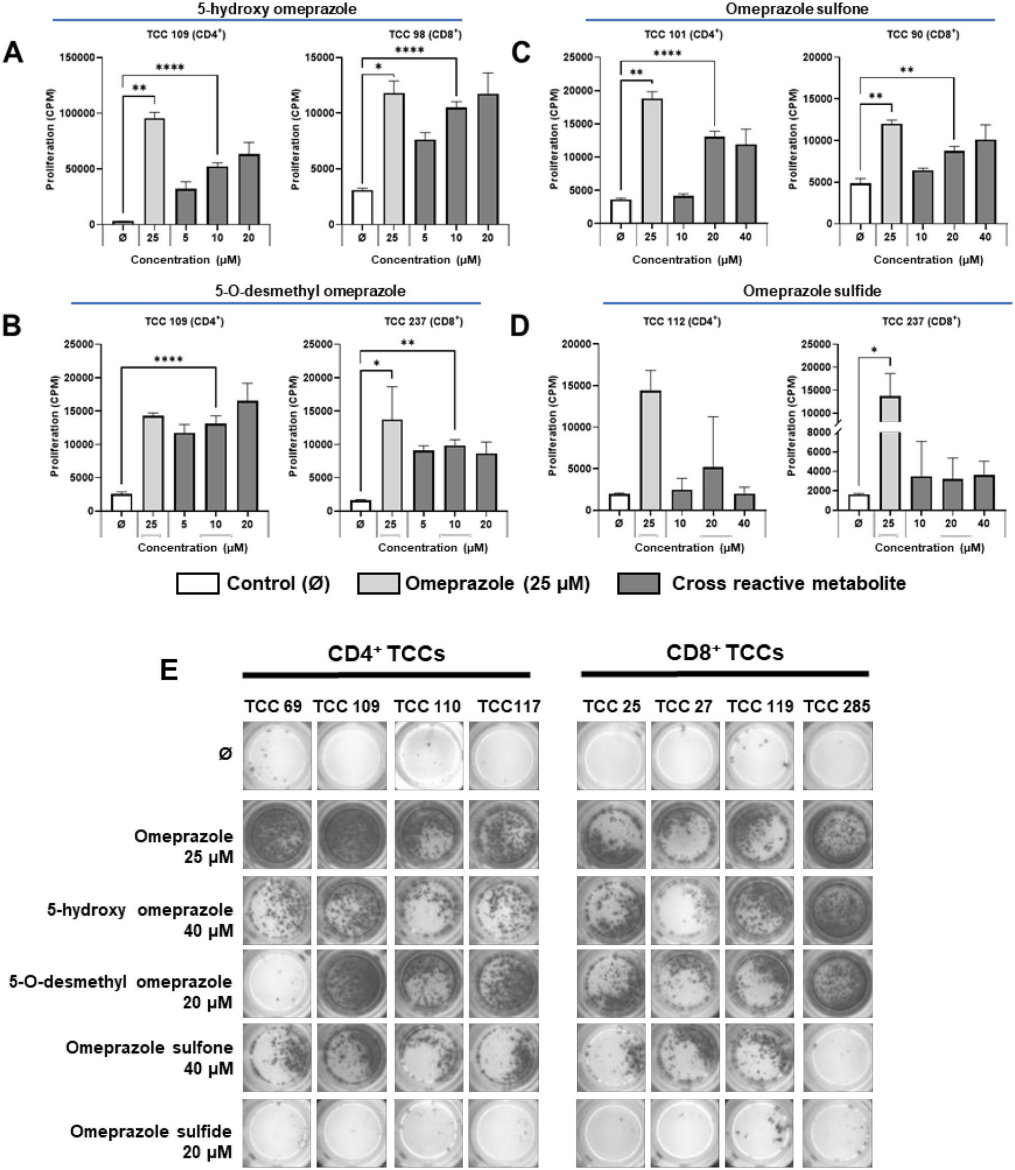
**CD4+ and CD8+ omeprazole-responsive T-cell clones display additional reactivity towards omeprazole metabolites**. CD4+ (*n* = 10) and CD8+ (*n* = 9) T-cell clones (5×10^4^) were cultured with irradiated autologous EBV-transformed B-cells (1×10^4^) and either **(A)** 5-hydroxy omeprazole, **(B)** 5-O-desmethyl omeprazole, **(C)** omeprazole sulfone, or **(D)** omeprazole sulfide. Proliferation was assessed after 48 h via [^3^H]-thymidine incorporation and is presented as CPM + SD. One representative CD4+ and CD8+ T-cell clone is presented for each compound. Statistical significance was determined using Dunnett’s multiple comparison 1-way ANOVA test for parametric data and using Dunn’s multiple comparison Kruskal-Wallis 1-way ANOVA test for nonparametric data (*P < 0.05, **P < 0.01, ***P < 0.001, ****P < 0.0001). **(E)** CD4+ (*n* = 12) and CD8+ (*n* = 9) T-cell clones (5×10^4^) were cultured with irradiated autologous EBV-transformed B-cells (1×10^4^) and test compounds, and IFN-γ secretion was assessed using ELISpot. Four representative CD4+ and CD8+ T-cell clones are presented.

Similar crossreactivity experiments were conducted with omeprazole metabolites; 5-hydroxy omeprazole, 5-O-desmethyl omeprazole, omeprazole sulfone, and omeprazole sulfide. CD4+ and CD8+ clones were stimulated to proliferate and secrete IFN-γ in the presence of 5-hydroxy omeprazole, 5-O-desmethyl omeprazole, and omeprazole sulfone (Fig. 4, Table 1), whereas significant responses were not detected with omeprazole sulfide.

### Activation of T-cell clones with omeprazole, omeprazole metabolites, and structurally related compounds required formation of antigen-presenting cell protein adducts

**Fig. 5.**
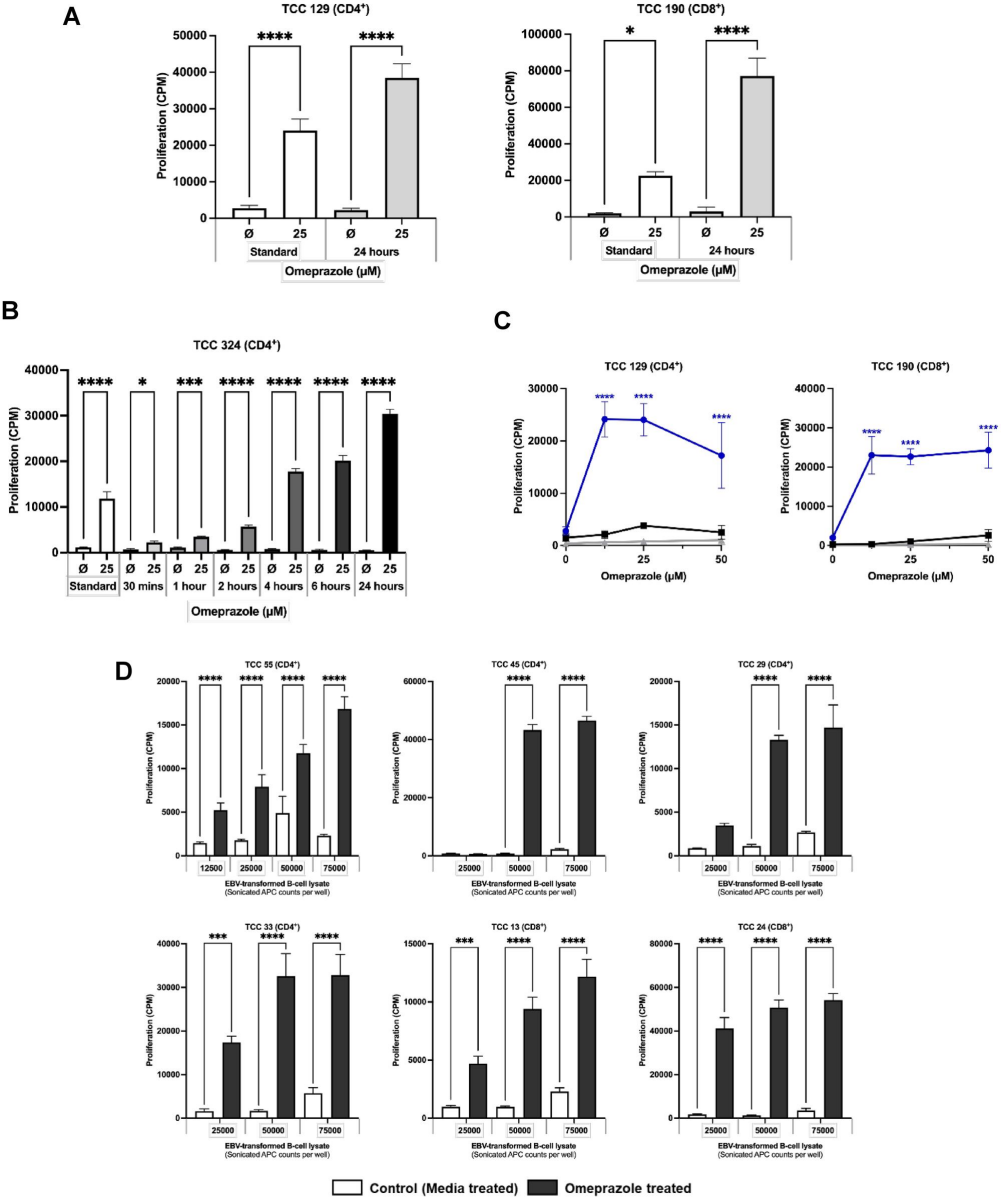
**Omeprazole T-cell clones are activated via a hapten pathway involving formation of antigen-presenting cell protein adducts. (A)** CD4+ (*n* = 15) and CD8+ (*n* = 6) T-cell clones (5×10^4^) were cultured with EBV-transformed B-cells (1×10^4^) and soluble omeprazole or with EBV-transformed B-cells (1×10^4^) pulsed with omeprazole for 24 h before repeated washing to remove unbound drug. One representative CD4+ and CD8+ T-cell clone is presented. **(B)** T-cell clones (*n* = 7) (5×10^4^) were cultured with EBV-transformed B-cells (1×10^4^) and soluble omeprazole or with omeprazole-pulsed EBV-transformed B-cells (1×10^4^) for 0.5 to 24 h. One representative CD4+ T-cell clone is presented. **(C)** CD4+ and CD8+ T-cell clones (*n* = 3) (5×10^4^) were cultured (i) alone with omeprazole (ii) with glutaraldehyde-fixed EBV-transformed B-cells (1×10^4^) and omeprazole or (iii) with standard EBV-transformed B-cell lines as APCs (1×10^4^) and omeprazole. **(D)** CD4+ and CD8+ T-cell clones (*n* = 3) (5×10^4^) were cultured with omeprazole-modified or unmodified EBV-transformed B-cell lysates. Proliferation was measured by addition of [^3^H]thymidine for the final 16 h of the culture period followed by scintillation counting. Results are presented as CPM + SD. Statistical significance was determined using 2-way ANOVA test (*P < 0.05, **P < 0.01, ***P < 0.001, ****P < 0.0001).

Antigen-presenting cells were initially pulsed with omeprazole for 24 h before repeated washing to remove unbound drug and culture with clones. All CD4+ and CD8+ clones were stimulated to proliferate with antigen-presenting cells pulsed with omeprazole (Fig. 5A). An extended pulsing experiment was subsequently performed to explore the kinetics of omeprazole antigen-presenting cell adduct formation and how this relates to T-cell activation. Clones were stimulated to proliferate weakly with antigen-presenting cells pulsed for 30 min and 1 h. The proliferative response of clones then increased in a time-dependent manner with longer antigen-presenting cell omeprazole culture periods (Fig. 5B). Antigen-presenting cells pulsed with omeprazole for 4-24 h stimulated higher levels of proliferation than soluble drug. Omeprazole did not activate T-cell clones in the absence of antigen-presenting cells. Furthermore, omeprazole-specific proliferation was not detected when antigen-presenting cells were fixed with glutaraldehyde to block antigen processing (Fig. 5C). Finally, lysates were prepared from antigen-presenting cells pulsed with omeprazole for 24 h and used as a source of antigen in assays with clones. The omeprazole-modified antigen-presenting cell lysates stimulated clones to proliferate in a protein concentration-dependent manner (Fig. 5D). Similar proliferative responses were not observed with lysates prepared from unmodified antigen-presenting cells. Collectively, these experiments demonstrate that T-cells are activated with omeprazole via a hapten mechanism involving the formation of antigen-presenting cell protein adducts.

**Fig. 6.**
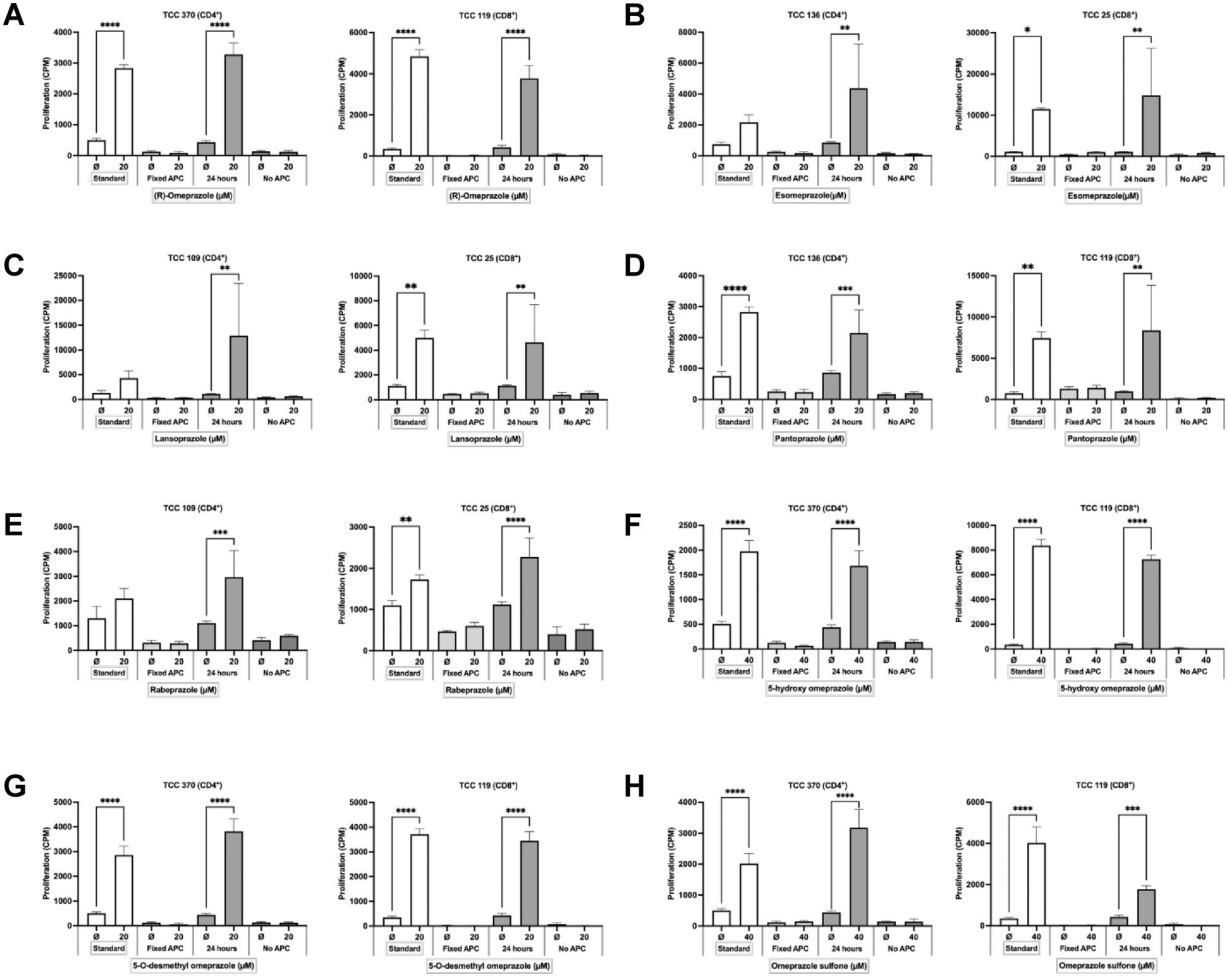
**Omeprazole T-cell clones are activated by (R)-omeprazole, proton pump inhibitors, and omeprazole metabolites by the hapten mechanism**. CD4+ (*n* = 3) and CD8+ (*n* = 3) T-cell clones (5×10^4^) were cultured with EBV-transformed B-cells (1×10^4^) and soluble **(A)** (R)-Omeprazole, **(B)** esomeprazole, **(C)** lansoprazole, **(D)** pantoprazole, **(E)** rabeprazole, **(F)** 5-hydroxy omeprazole, **(G)** 5-O-desmethyl omeprazole, and **(H)** omeprazole sulfone or with drug metabolite-pulsed EBV-transformed B-cells (1×10^4^) for 1 and 24 h. One representative CD4+ and CD8+ T-cell clone is presented. Proliferation was measured via [^3^H]-thymidine incorporation and is presented as CPM + SD. One representative CD4+ and CD8+ T-cell clone is presented. Statistical significance was determined using 2-way ANOVA test (*P < 0.05, **P < 0.01, ***P < 0.001, ****P < 0.0001).

Similar experiments were conducted with alternative proton pump inhibitors previously identified to stimulate omeprazole-responsive T-cells as outlined in Fig. 3 and omeprazole metabolites. For all compounds, (i) antigen-presenting cells were required for T-cell activation, (ii) fixation of antigen-presenting cells prevented compound-specific activation of T-cells, and (iii) an extended 24 h antigen-presenting cell culture period generated T-cell stimulatory drug-protein adducts (Fig. 6).

### T-cell stimulatory proton pump inhibitors and omeprazole metabolites form adducts with off-target proteins

**Fig. 7.**
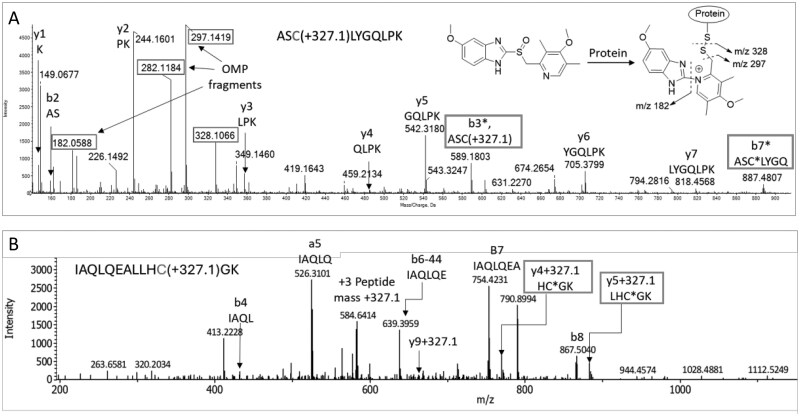
**Characterization of omeprazole-protein adduct formed in vitro**. Overnight incubation of omeprazole with GSTP or EBV-transformed B cell lines was carried out followed by tryptic digestion and C18 resin clean up. (A) Representative MS/MS spectrum of a triply charged ion corresponding to GSTP peptide ^45^ASC[Omeprazole]LYGQLPK^54^ modified at Cys47 with omeprazole. Peptide fragments are indicated at b and y ions and boxed in red is the fragments derived from omeprazole and fragment ions containing omeprazole modification are boxed in green. (B) Representative MS/MS spectra of a triply charged ion at m/z 584.3033 corresponding to an omeprazole-modified peptide (^5336^IAQLQEALLHCGK^5348^) derived from microtubule-actin cross-linking factor 1 (Q9UPN3|MACF1_HUMAN). Fragment ions containing omeprazole modification are boxed in green.

To assess the protein reactivity of omeprazole, proton pump inhibitors, and omeprazole metabolites, the compounds were incubated with the protein GSTP for 24 h. GSTP contains a reactive cysteine residue (Cys47) that is easily modifiable with electrophilic drugs (Meng et al. 2016). Figure 7A depicts the MS/MS spectrum for a triply charged ion at m/z 469.5 corresponding to ^45^ASCLYGQLPK^54^ with a mass addition of 327.11 which corresponds to omeprazole bound covalently to GSTP. Figure S4 shows comparable MS/MS spectrum with esomeprazole, lansoprazole, pantoprazole, and rabeprazole bound to the cysteine group in same peptide of GSTP following 24 h incubation. Similar GSTP adducts were detected on peptide ^45^ASCLYGQLPK^54^ with 5-hydroxy and 5-O-desmethyl omeprazole (Fig. S5). In contrast, GSTP did not form detectable adducts with omeprazole sulfone and omeprazole sulfide (data not shown).

To identify omeprazole-modified off-target intracellular proteins that are potentially antigens responsible for T-cell activation, EBV-transformed B-cell lines were incubated with omeprazole for 24 h. Mass spectrometric analysis of the tryptic digests of cell lysates revealed over 30 omeprazole-modified peptides derived from 36 proteins (Table S1). Figure 6B shows MS/MS spectra of a triply charged ion at m/z 584.3033 corresponding to an omeprazole-modified peptide derived from Microtubule-actin cross-linking factor 1 (Q9UPN3|MACF1_HUMAN: O94854|K0754_HUMAN) ^5336^IAQLQEALLHCGK^5348^. A series of y ions with a mass addition of 327.13 suggests omeprazole is conjugated to Cys5346 (Fig. 7B). A diverse range of proteins with different functions were found to be targeted by omeprazole, including microtubule-binding motor proteins (e.g. Kinesin heavy chains and microtubule-actin cross-linking factor 1), membrane traffic proteins (e.g. Golgin subfamily A member 6), and proteins with catalytic activities (Pyruvate kinase PKM, protein disulfide-isomerase, and Transcription activator BRG1) (Fig. S6). There is limited data describing expression of H+/K+ ATpase expression in B-cells. It was not detected in our mass spectrometry experiments.

## Discussion

Given that covalent inhibition of protein function has seen a resurgence within Pharma as a therapeutic mode of action, there is an urgent need to characterize off-target binding interactions and to understand whether this represents an immunological risk for patients. Targeted covalent inhibitors exert pharmacological function in 2 steps: First, through reversible interactions with the protein target (which infers selectivity); and secondly, through covalent ligation of a specific amino acid that the drug has been brought into close orientation with (which infers potency) (Baillie 2021). The selective nature of the noncovalent drug target protein interaction in principle limits the extent of off-target binding and hence may mitigate the risk of immune-mediated adverse events; however, no one drug can display exquisite binding selectivity towards one protein. Thus, this study aimed to investigate whether off-target covalent drug protein binding perceived as immunogenic, exploring the relationship between adducts formed in antigen-presenting cells and immune recognition?

Omeprazole is a pro-drug converted under acid conditions to a reactive sulfenamide that binds protein irreversibly by forming disulfide linkages with cysteine residues within parietal cell H+/K+ ATPases. It is rapidly absorbed after oral administration, with peak plasma concentrations typically occurring in 0.5 to 3.5 h (Mihaly et al. 1983). Peak plasma concentrations of omeprazole and its metabolites are in the low microM range, which have been shown to active patient PBMC (Grice et al. 2024). Thus, omeprazole is well placed as a candidate to explore whether off-target protein binding generates a molecular initiating event for T-cell activation. Previous studies have shown using antidrug antibodies that omeprazole forms adducts with multiple intracellular proteins, but these adducts were only observed at high mM drug concentrations and the site and nature of the modifications were not defined (Cartee and Wang 2020).

Herein, mass spectrometry was used to characterize the proteins modified within antigen-presenting cells by omeprazole. Omeprazole-responsive T-cell clones were then used in a range of functional assays to demonstrate that peptides derived from drug-modified antigen-presenting cell protein are immunogenic. Omeprazole is metabolized in the liver by cytochrome P450 enzymes CYP2C19 and CYP3A4 forming the major metabolites 5-hydroxy omeprazole and omeprazole sulfone, and lesser metabolites 5-O-desmethyl omeprazole, 3-hydroxyomeprazole, omeprazole sulfone, and omeprazole sulfide. Thus, the model protein GSTP was used to investigate the binding profile of omeprazole metabolites and then explore the relationship between metabolite protein binding and activation of T-cells. Clinical crossreactivity has been reported between PPIs (Caboni et al. 2007; Cox and Kaplan 2007; Bavbek et al. 2024), which is thought to relate to structural similarities (they each contain a benzimidazole and a pyridine ring). Thus, activation of omeprazole-responsive clones with different PPIs and alternative H2 antagonists was also assessed.

CD4+ and CD8+ T-cells were stimulated to proliferate and release cytokines and cytolytic molecules (e.g. IFN-γ, IL-5, IL-13, TNF-α, IL-10, IL-6, IL-22, perforin, granzyme B, and sFasL) in the presence of omeprazole, providing evidence that the immune cells were polyfunctional. CD4+ and CD8+ clones secreted Th1 and Th2 cytokines and cytolytic molecules; however, noticeable differences were observed. TNF-α, perforin, granzyme B, and sFas-L were preferentially secreted from omeprazole-treated CD8+ T-cells. In contrast, CD4+ T-cell clones secreted higher levels of the cytokines IL-5, 6, and 22. Omeprazole-specific activation of the clones was dependent on the presence of antigen-presenting cells (EBV-transformed B-cells), whereas antibody blocking experiments demonstrated that the response of CD4+ and CD8+ clones was MHC class II and MHC class I restricted. Antigen-presenting cell pulsing assays, where noncovalently bound drug is removed from antigen-presenting cells prior to the addition of T-cells (Castrejon et al. 2010), were used to determine whether clones were activated with soluble drug bound reversibly to MHC peptide complexes or hapten protein conjugates. All clones were stimulated to proliferate strongly with antigen-presenting cells pulsed for 4-24 h, indicating that (i) covalent binding of omeprazole to antigen-presenting cell protein generates antigenic determinants to activate T-cells, and (ii) adduct formation was time dependent. Fixation of antigen-presenting cells, which inhibits antigen processing, but not the direct binding of drugs to MHC displayed on the cell surface (Zanni et al. 1998), blocked the activation of clones with omeprazole. Thus, direct (covalent or noncovalent) binding of omeprazole to peptides displayed on surface MHC molecules does not activate T-cells. Given that omeprazole covalent binding within antigen-presenting cells is a requisite for the activation of clones, omeprazole-modified antigen-presenting cell lysates were generated and used as a source of antigen. Clones were stimulated to proliferate with the cell lysates in a concentration-dependent manner and the strength of the proliferative response was similar to that observed with soluble drug. Although detailed investigation of pathways of antigen processing and presentation was beyond the scope of the study, it is likely that omeprazole-responsive CD8+ cells are activated with intracellular antigens loaded directly onto MHC class I molecules in the endoplasmic reticulum and through cross presentation, whereas CD4+ cells are activated adducts are engulfed by antigen-presenting cells and enter the MHC class II processing pathway. B-cells were used as antigen-presenting cells to initially test clones and in the functional assays. B-cells are efficient at presenting antigens to CD4+ and CD8+ memory T-cells (i.e. drug-responsive T-cells from patients with adverse reactions) but are less efficient at priming naïve T-cells. This avoided the impact of potentially confounding co-stimulatory signaling associated with professional antigen-presenting cells (e.g. dendritic cells) in our in vitro assay. Mass spectrometric analysis revealed modification of 36 proteins by omeprazole, supporting the hypothesis that omeprazole activates T-cells via covalent binding to antigen-presenting cells. Beyond generating drug antigens, covalent binding of omeprazole to those proteins with catalytic functions can potentially alter their downstream activities, leading to cellular stress. Of particular interest is protein-disulfide isomerase (PDI), a key enzyme found in the endoplasmic reticulum and on the cell surface, which facilitates oxidative protein folding and prevents protein misfolding/aggregation (Wang et al. 2012). Although omeprazole was found to bind to Cys343, a residue outside PDI’s redox-active domain, this modification could still influence its reducing potential (González-Perilli et al. 2017). Notably, the highly reactive cysteines at the active sites (Cys53 and Cys397) are prone to unstable covalent modifications, making them harder to detect (Marttila et al. 2014). Whether the omeprazole protein adducts persist in the long term is not known and beyond the scope of our study. Cartee and Wang (2020) showed that omeprazole is capable of forming highly stable complexes that are not dependent on disulfide linkages. Thus, an investigation of the adducts formed in patients, their stability, and importance in T-cell activation warrants further investigation.

Sulfa drugs such as dapsone and sulfamethoxazole have been used as paradigms to define the chemical basis of drug-specific T-cell activation due to their well-defined metabolism and the availability of clinical samples from patients with adverse reactions (Schnyder et al. 1997, 2000; Burkhart et al. 2001; Castrejon et al. 2010; Elsheikh et al. 2011; Zhao et al. 2019, 2021; Almutairi et al. 2023). CD4+ and CD8+ T-cells are activated with the parent drug through direct HLA-peptide T-cell receptor binding (Schnyder et al. 1997; Castrejon et al. 2010; Zhao et al. 2019). Closely related stable metabolites and drug derivatives activate the same T-cells through crossreactivity, whereas more extensive structural modifications such as the addition of an acetyl group prevents T-cell activation (von Greyerz et al. 1999; Depta et al. 2004; Zhao et al. 2019), presumably by disrupting the HLA-peptide T-cell receptor binding interaction. Dapsone and sulfamethoxazole are also metabolized to reactive nitroso metabolites that bind to cysteine residues on protein generating hapten protein conjugates (Naisbitt et al. 1996, 1999), that activate discrete T-cells in the same hypersensitive patients (Schnyder et al. 2000; Castrejon et al. 2010; Zhao et al. 2019; Almutairi et al. 2023). Although beyond the scope of the current study, we are using sulfa drugs and omeprazole to explore (i) the nature of the omeprazole-modified peptides displayed naturally by MHC class I and II molecules expressed on antigen-presenting cells and (ii) which of these peptides activate CD4+ and CD8+ T-cells.

Omeprazole exists as a mixture of 2 stereoisomers (R)-omeprazole and (S)-omeprazole, whereas esomeprazole is the pure S enantiomer of omeprazole (Cederberg et al. 1989). Other proton pump inhibitors (lansoprazole, pantoprazole, rabeprazole) contain similar ring structures connected through a sulfoxide moiety. Thus, crossreactivity studies were conducted to see how the differing side chains impact the omeprazole-specific T-cell response. Almost 100% of CD4+ and CD8+ TCC were activated with both pure stereoisomers of omeprazole, which is similar to the activation of T-cells observed with both enantiomeric forms of the protease inhibitor telaprevir (Alhilali et al. 2019). Other proton pump inhibitors stimulated between 30% and 60% of the clones to proliferate and secrete IFN-γ. All the proton pump inhibitors activated clones via a hapten mechanism with an antigen-presenting cell culture time of 24 h optimal for the formation of T-cell stimulatory protein adducts. Each drug was also shown to covalently modify the highly reactive Cys^47^ residue within the ^45^ASCLYGQLPK^54^ peptide found in GSTP. This provides direct evidence that all proton pump inhibitors bind to off target proteins forming immunogenic drug-protein adducts. In contrast, structurally dissimilar H2 antagonists, which also block gastric acid secretion, did not activate the omeprazole-responsive clones. The data provide evidence that the structurally related compounds generate shared antigenic determinants, but it remains unclear whether T cells recognize common haptenated motifs, distinct adducts presented on similar peptides, or whether some degree of TCR promiscuity contributes to the observed responses.

Crossreactivity studies also revealed that T-cell clones were activated with omeprazole metabolites. Particularly strong responses were detected in the presence of 5-hydroxy omeprazole, 5-O-desmethyl omeprazole, and omeprazole sulfone, where almost all TCC were stimulated to proliferate and secrete cytokines. Clones were again activated via a hapten pathway involving formation of antigen-presenting cell metabolite protein adducts, whereas inhibition of antigen processing blocked T-cell activation with the drug metabolites. 5-Hydroxyomeprazole and 5-O-desmethyl omeprazole formed covalent adducts with Cys^47^ on the ^45^ASCLYGQLPK^54^ peptide found in GSTP. Collectively, these data indicate that although the metabolites are deemed pharmacologically inactive, they may still be immunologically active through generation of antigen-presenting cell protein adducts similar to omeprazole. In contrast, very low numbers of T-cells were activated with omeprazole sulfide, and the strength of the induced proliferative response was weak. This could be due to the absence of a sulfoxide structure which may prevent the metabolite from forming a reactive sulfenamide. In support of this, omeprazole sulfide GSTpi adducts were not detectable by mass spectrometry. Finally, omeprazole sulfone activated T-cells via a hapten pathway, but adducts with GSTP were not detected. It is possible that adducts are formed with GSTP below the limits of detection or more likely the sulfone metabolite binds to alternative proteins within antigen-presenting cells.

It is interesting to speculate why despite extensive prescription and use of omeprazole the occurrence of hypersensitivity reactions is rare and less than with other covalent binding drugs such as β-lactam antibiotics. There are several possible explanations for this: (i) differences in the absolute levels of drug binding with omeprazole forming lower levels of adducts, (ii) differences in the numbers of proteins modified with omeprazole conjugating fewer proteins and hence generating lower levels of antigen for HLA binding, (iii) differences in HLA proteins that present the drug antigens, with omeprazole reactions possibly displaying a higher degree of HLA allele restriction, and (iv) differences with immune (dys)regulation. Patients are administered antibiotics to treat infection and as such the innate immune response and “danger signaling” at the time of drug exposure may be skewed and favor drug-specific T-cell activation.

In conclusion, our data provide evidence that omeprazole causes T-cell-mediated hypersensitivity reactions and omeprazole should be viewed as a potential causative agent when reactions present in patients exposed to multiple drugs. Extensive crossreactivity with structurally related proton pump inhibitors highlights that careful consideration must be exercised when administering alternative drugs to patients presenting with hypersensitivity. Omeprazole activated clones via a hapten mechanism and a direct relationship between off-target protein binding and T-cell activation was observed. Targeted covalent inhibitors designed for cancer therapy cause skin reactions and, to a lesser extent, hepatotoxicity which are likely to be caused via a similar pathway through covalent modification off-target proteins.

## Supplementary Material

kfag046_Supplementary_Data
