# Supplementary material for: Omeprazole activation of CD4+ and CD8+ T-cells through off-target covalent modification of cellular proteins
Source: Toxicol Sci. 2026 Apr 27;209(5):kfag046. doi: 10.1093/toxsci/kfag046 (PMC13167194; doi:10.1093/toxsci/kfag046)
Supplement: kfag046_Supplementary_Data [file kfag046_supplementary_data.docx]

**Title: Omeprazole activation of CD4+ and CD8+ T-cells through off-target covalent modification of cellular proteins**

**Running title:** T-cell activation by drug haptens

**Authors:** Sophie Grice^1^, Sa’d Albashtawy^1^, Georgia Wells^1^, Luisa Hering^1^, Kareena Adair^2^, Joscelyn Sarsby^2^, Philip Brownridge^2^, Megan Ford^1^, Rachel Lloyd^1^, Lucy Hampson^1^, Annette Wagner^3^, Yonghu Sun^4^, Hong Liu^4^, Sean Hammond^1,5^, Xiaoli Meng^1^, Furen Zhang^4^, Dean Naisbitt^1*^.

*^1^Department of Pharmacology and Therapeutics, University of Liverpool, United Kingdom.*

*^2^ Centre for Proteome Research, University of Liverpool, United Kingdom.*

*^3^* *Department of Adult Allergy, Guy’s and St Thomas’ Hospital, London, UK*

*^4^ Shandong Provincial Institute of Dermatology and Venereology, Shandong Academy of Medical Sciences, Jinan, Shandong, China.*

*^5^* *ApconiX, Alderley Edge SK10 4TG, UK*

**Key words:** Targeted covalent inhibitor drugs, omeprazole, T-lymphocytes, hapten, crossreactivity, allergy.

***Corresponding author:** Professor Dean Naisbitt, Department of Pharmacology and Therapeutics, Sherrington Building, Ashton Street, The University of Liverpool, Liverpool L69 3GE, England, Telephone: +44 151 7945346. E-mail: dnes@liverpool.ac.uk

**Supplementary Figures**

**Supplementary Figure 1: CD4+ and CD8+ omeprazole T-cell clones secrete effector cytokines and cytolytic molecules. (A)** CD4+ (n=4) and **(B)** CD8+ (n=4) T-cell clones (5x10^4^) were cultured with irradiated autologous EBV transformed B-cells (1x10^4^) and omeprazole. Secretion of IL-5, IL-13, IL-17, IL-22, granzyme B and perforin was assessed using ELISpot.

**Supplementary Figure 2: Activation of CD8+ and CD4+ T-cell clones with omeprazole was MHC class I and MHC class II restricted.** CD4+ (n=3) and CD8+ (n=3) T-cell clones (5x10^4^) were incubated with omeprazole and irradiated autologous EBV transformed B-cells (1x10^4^). MHC blocking antibodies were added 15 min before omeprazole. Proliferation was measured via [^3^H]-thymidine proliferation and data are presented as CPM + standard deviation. Statistical significance was determined using two-way ANOVA test (*p<0.05, **p<0.01, ***p< 0.001, ****p<0.0001).

**Supplementary Figure 3: Cross-reactivity of omeprazole-responsive T-cell clones with H2 antagonists.** T-cell clones (n=3) (5x10^4^) were incubated with irradiated autologous EBV transformed B-cells (1x10^4^) and omeprazole, famotidine or nizatidine. Proliferation was measured via [^3^H]-thymidine incorporation and is presented as CPM + standard deviation. Statistical significance was determined using Dunnett's multiple comparison one-way ANOVA test for parametric data and using Dunn’s multiple comparison Kruskal-Wallis one-way ANOVA test for non-parametric data (*p<0.05, **p<0.01, ***p< 0.001, ****p<0.0001).

**Supplementary Figure 4: Characterisation of esomeprazole-GSTP, lansoprazole-GSTP, pantoprazole-GSTP and rabeprazole-GSTP adducts formed in vitro.** Incubation of **(A)** esomeprazole, **(B)** lansoprazole, **(C)** pantoprazole and **(D)** rabeprazole with GSTP was carried out followed by tryptic digestion of the sample. This was followed by C18 resin clean up before samples were analyzed on a Sciex TripleTOF 6600 mass spectrometer. **(A)** Representative MS/MS spectra of a triply charged ion corresponding to GSTP peptide ^45^ASC[Esomeprazole]LYGQLPK^54^ modified at Cys47 with esomeprazole. Peptide fragments are indicated at b and y ions and boxed in red is the esomeprazole modification on the fragment ion b_3_ labelled 589.1790. **(B)** Representative MS/MS spectra of a triply charged ion corresponding to GSTP peptide ^45^ASC[Lansoprazole]LYGQLPK^54^ modified at Cys47 with lansoprazole. Peptide fragments are indicated at b and y ions and boxed in red is the lansoprazole modification on the fragment ion b_3_ labelled 613.1409. **(C)** Representative MS/MS spectra of a triply charged ion corresponding to GSTP peptide ^45^ASC[Pantoprazole]LYGQLPK^54^ modified at Cys47 with pantoprazole. Peptide fragments are indicated at b and y ions and boxed in red is the pantoprazole modification on the fragment ion b_3_ labelled 627.1331. **(D)** Representative MS/MS spectra of a triply charged ion corresponding to GSTP peptide ^45^ASC[Rabeprazole]LYGQLPK^54^ modified at Cys47 with rabeprazole. Peptide fragments are indicated at b and y ions and boxed in red is the rabeprazole modification on the fragment ion b_3_ labelled 603.1954.

**Supplementary Figure 5: Characterisation of 5-hydroxy omeprazole-GSTP and 5-O-desmethyl omeprazole-GSTP adduct formed in vitro**. Incubation of **(A)** 5-hydroxy omeprazole and **(B)** 5-O-desmethyl omeprazole with GSTP was carried out followed by tryptic digestion of the sample. This was followed by C18 resin clean up before samples were analyzed on a Sciex TripleTOF 6600 mass spectrometer. **(A)** Representative MS/MS spectra of a triply charged ion corresponding to GSTP peptide ^45^ASC[5-hydroxy omeprazole]LYGQLPK^54^ modified at Cys47 with 5-hydroxy omeprazole. Peptide fragments are indicated at b and y ions and boxed in red is the pantoprazole modification on the fragment ion b_3_ labelled 605.1747. **(B)** Representative MS/MS spectra of a triply charged ion corresponding to GSTP peptide ^45^ASC[5-O-desmethyl omeprazole]LYGQLPK^54^ modified at Cys47 with 5-O-desmethyl omeprazole. Peptide fragments are indicated at b and y ions and boxed in red is the rabeprazole modification on the fragment ion b_3_ labelled 575.1644.

**Supplementary Figure 6: Omeprazole-modified proteins detected in antigen presenting cells.** A diverse range of proteins with different functions were found to be targeted by omeprazole. Protein class, molecular function and biological process were analysed using panther 19.0.

**
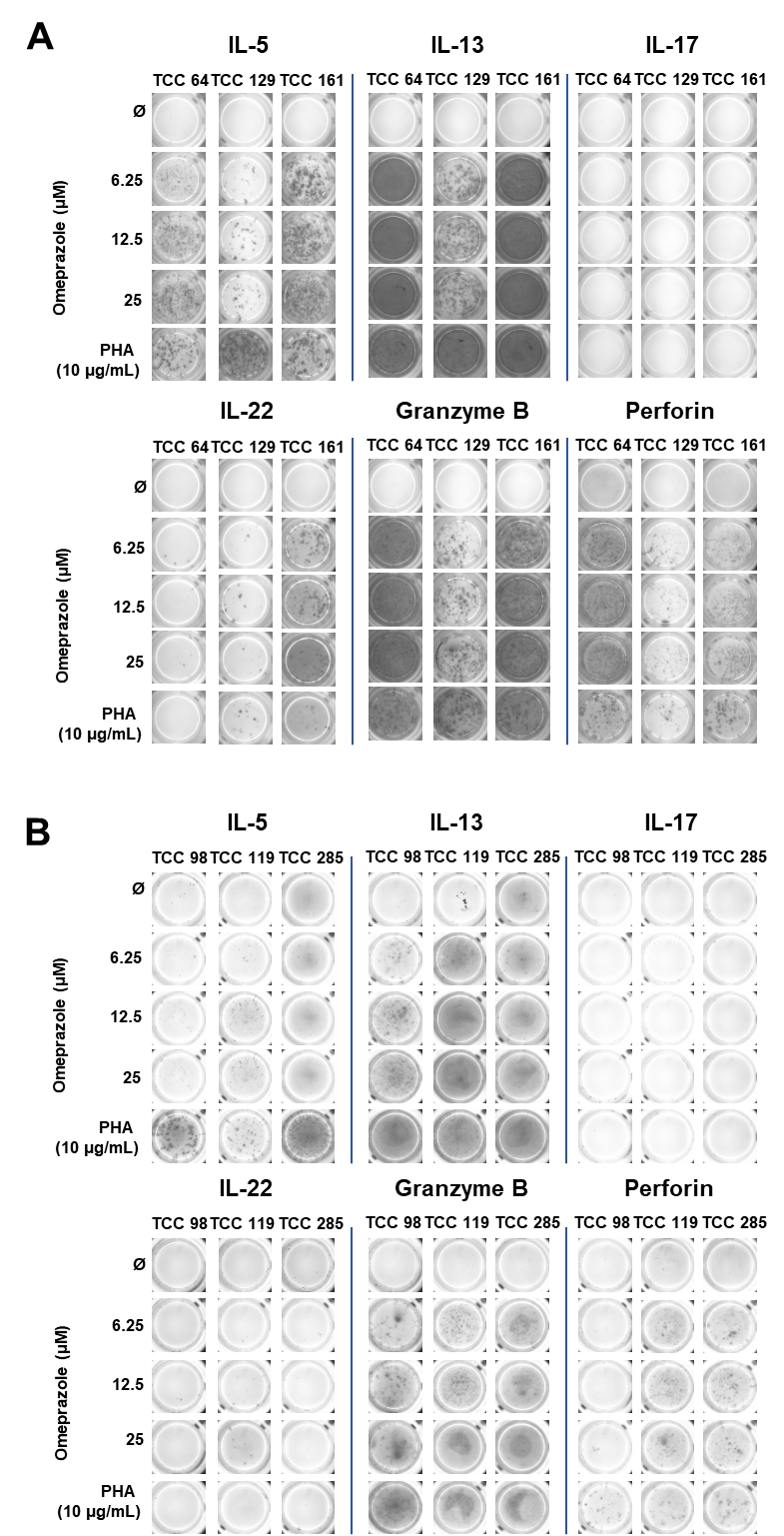
Supplementary Figure 1**

**Supplementary Figure 2**

**
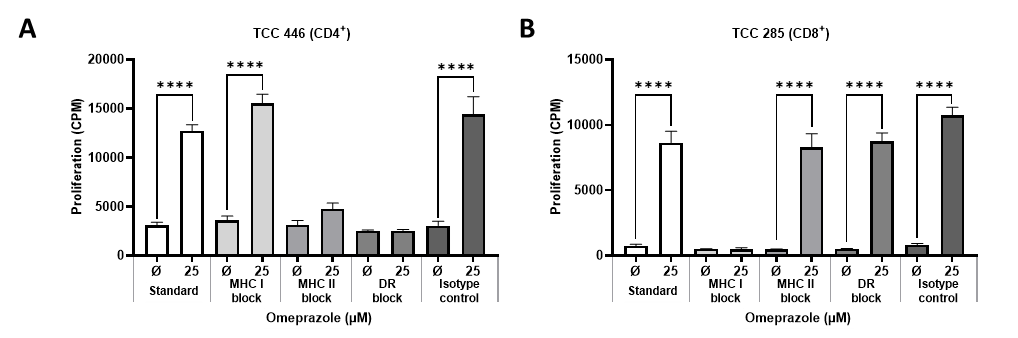
**

**
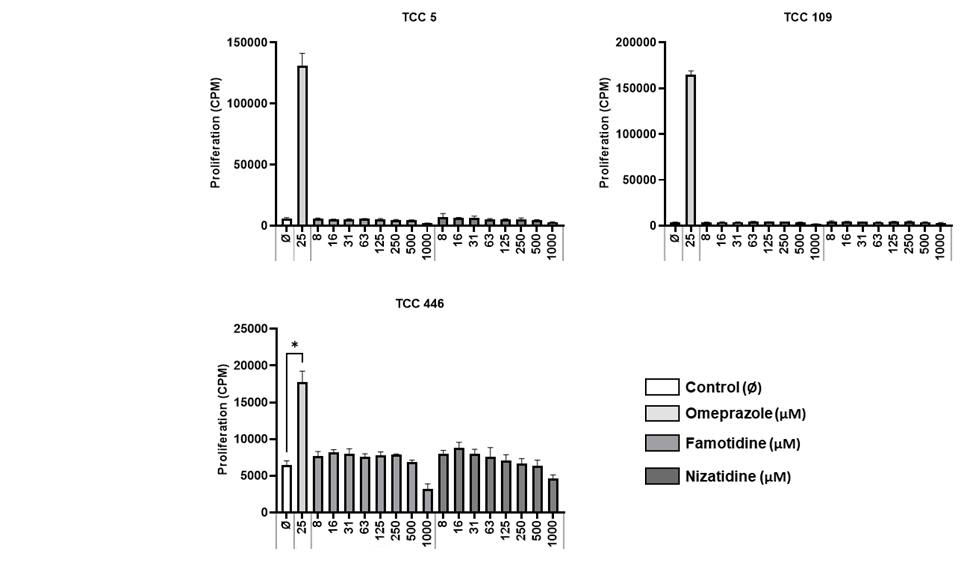
Supplementary Figure 3**

**Supplementary Figure 4**

**
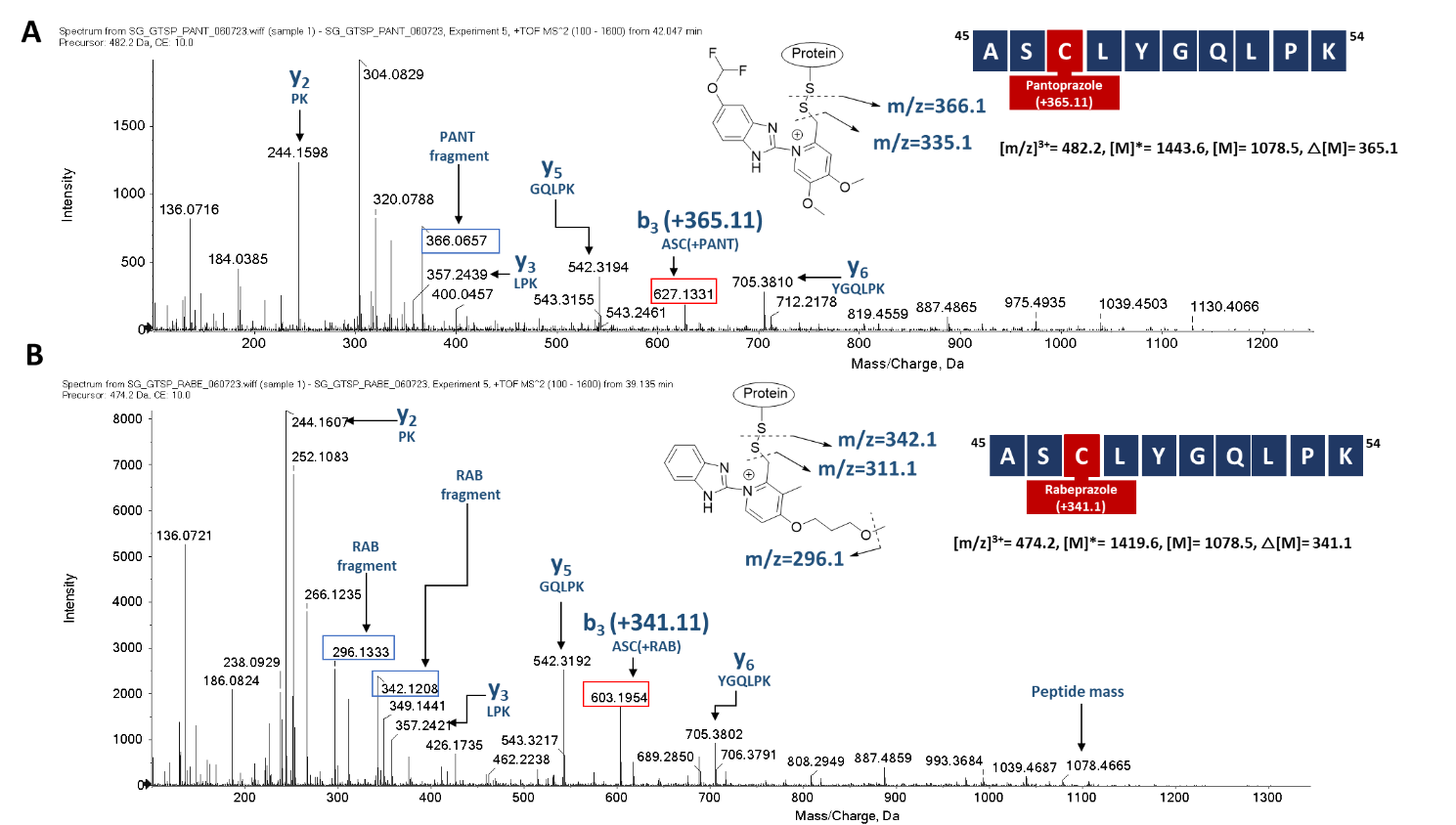
**


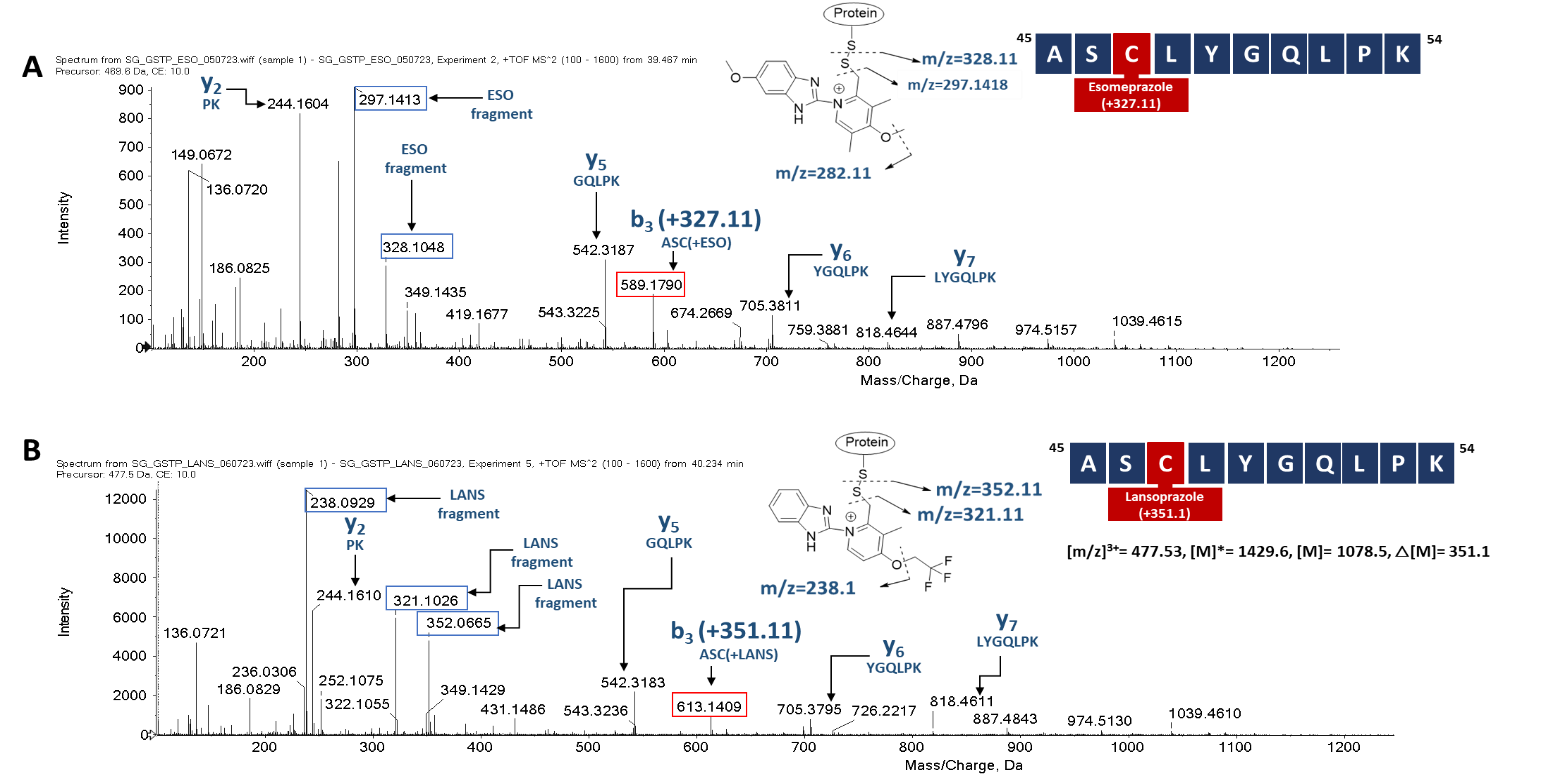


**[m/z]^3+^ = 469.6, [M]* = 1405.61, [M] = 1078.5, △[M] = 327.11**

**A**

**B**

**D**

**C**

**
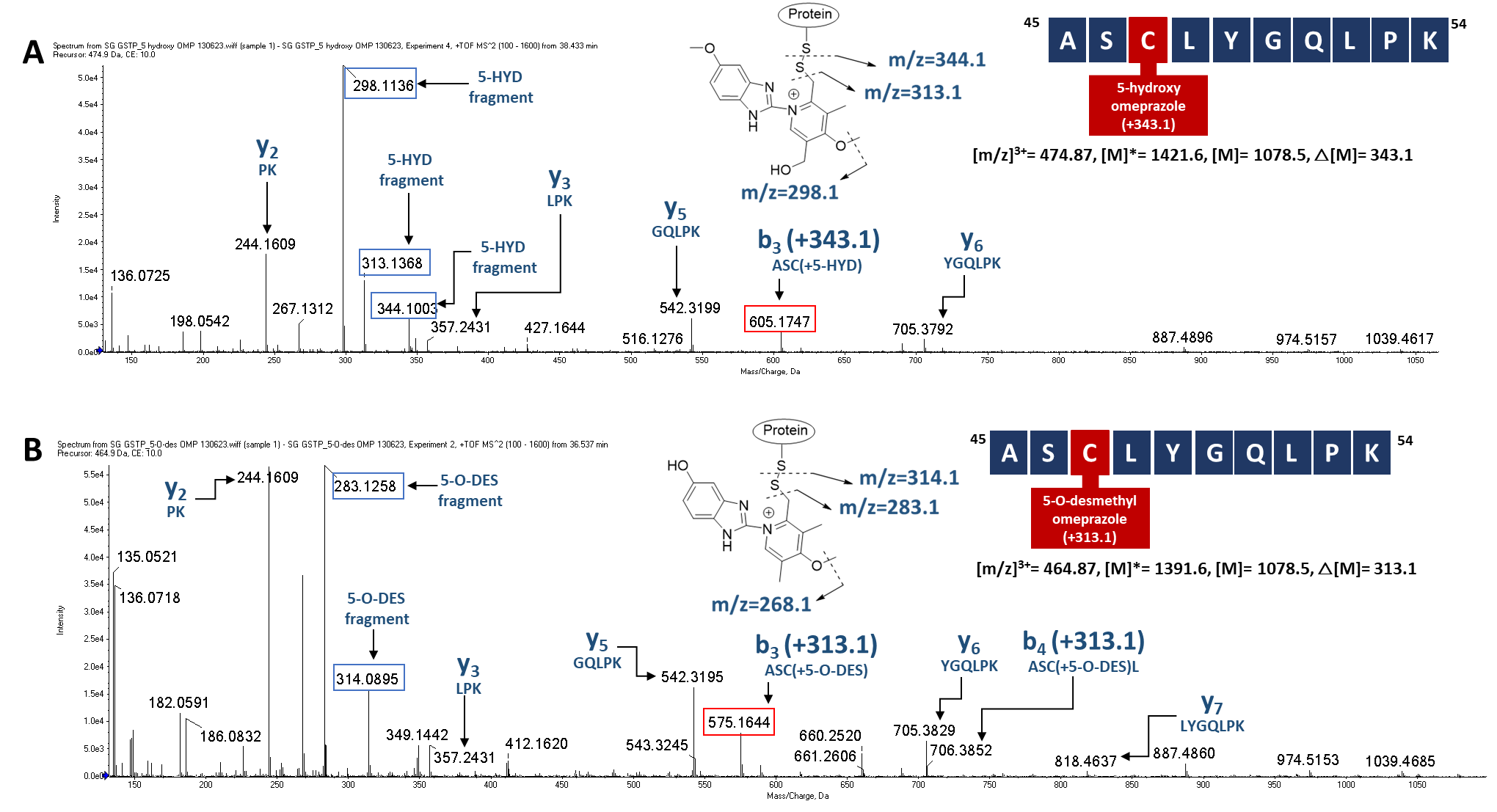
Supplementary Figure 5**

**Supplementary Figure 6**

**
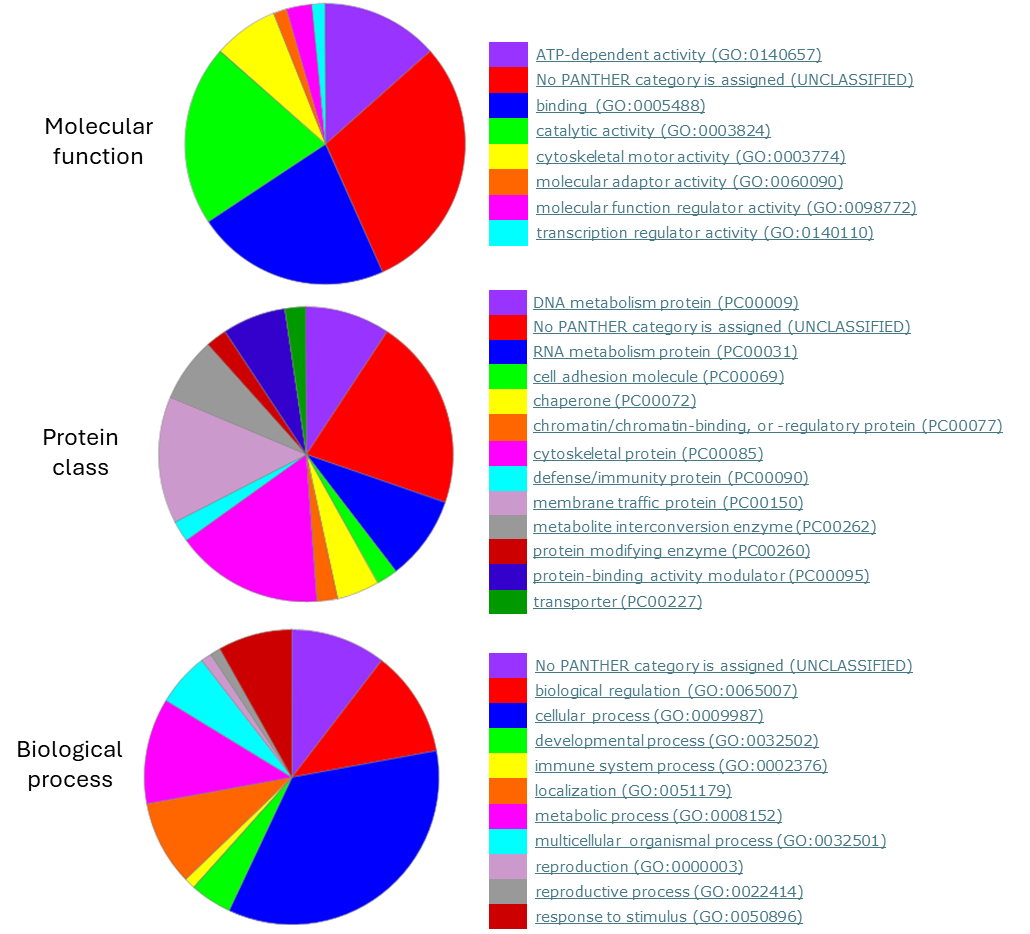
**

**Supplementary Table 1. Omeprazole-modified proteins detected in antigen presenting cells treated with omeprazole. EBV-transformed B-cells from the hypersensitive patient were treated with 100 µM omeprazole for 24 hours prior to lysis; cell lysate was digested with trypsin and analysed by LC-MS/MS. Peptide sequences were determined by PEAKS X pro 10.6 (Bioinformatics Solution Inc), * indicates omeprazole modified residue.**

| **Peptide** | **Amino acid** | **-10LgP** | **Mass** | **RT** | **Accession** |
| --- | --- | --- | --- | --- | --- |
| AAFGLSEAGFNTAC*VTKLFPTR | C89 | 22.3 | 2627.266 | 34.9 | P31040\|SDHA_HUMAN |
| AEGSDVANAVLDGADC*IM(+15.99)LSGETAK | C358 | 25.96 | 2779.214 | 33.1 | P14618\|KPYM_HUMAN |
| AQAGVVGEVTQVLC*AAGGALELPELRRR | C15 | 20.05 | 3189.654 | 33.8 | Q9H0J9\|PAR12_HUMAN |
| DMHGLFDVEISLTVQENAGSISC*SMR | C206 | 20.96 | 3165.403 | 36.2 | Q6UX41\|BTNL8_HUMAN |
| EAHLPPGAMAAVGLSWEEC*KQR | C630 | 24.03 | 2706.251 | 21.3 | P49327\|FAS_HUMAN |
| ENQNEAKENSASC*VENNIENIYGDK | C491 | 22.82 | 3138.329 | 30.2 | Q9BXT5\|TEX15_HUMAN |
| FHFMSPPPAQSMVQALELLYALGGLDKDC*RLTEPLGM(+15.99)R | C473 | 21.3 | 4574.208 | 40.4 | Q9H5Z1\|DHX35_HUMAN |
| FSEDTAEGEEANTYESHLLVC*FLKR | C485 | 23.48 | 3214.438 | 32.6 | Q96NR3\|PTHD1_HUMAN |
| GLALLC(+57.02)ALLGTLC*ETGSGQIR | C24 | 25.15 | 2472.233 | 31.5 | Q9Y5H0\|PCDG3_HUMAN |
| GPFSEISAFKTC(+57.02)LPGFPGAPC*AIK | C1895 | 23.47 | 2821.343 | 30.4 | P51610\|HCFC1_HUMAN |
| IAQLQEALLHC*GK | C5346 | 24.6 | 1749.869 | 34.8 | Q9UPN3\|MACF1_HUMAN:O94854\|K0754_HUMAN |
| KVGWPGESC*WQVGLAVEDSPALGAPR | C10 | 21.95 | 3035.442 | 31.2 | O95398\|RPGF3_HUMAN |
| LC*TMPPVGTDLNTVK | C6137 | 21.76 | 1914.904 | 23.8 | Q9UPN3\|MACF1_HUMAN:O94854\|K0754_HUMAN |
| LKAMIMC*KGC(+57.02)GAFC(+57.02)HDDC(+57.02)IGPSK | C1410 | 24.97 | 2925.239 | 15.6 | Q76L83\|ASXL2_HUMAN |
| LQLGM(+15.99)LQQAQC*GLDLRHITVVELVGVFPTLIGR | C367 | 20.51 | 3960.094 | 39.8 | P78539\|SRPX_HUMAN |
| LTC*EEEEEK | C1296 | 28.21 | 1435.564 | 5.0 | P51531\|SMCA2_HUMAN:P51532\|SMCA4_HUMAN |
| M(+15.99)TRILQDSLGGNC*R | C296 | 27.77 | 1905.865 | 16.8 | O60282\|KIF5C_HUMAN:P33176\|KINH_HUMAN:Q12840\|KIF5A_HUMAN |
| MADHVQSLAQLENLC*KQLYETTDTTTR | C15 | 20.94 | 3435.59 | 31.4 | Q9UIA9\|XPO7_HUMAN |
| NDDVKC*FC*C(+57.02)DGGLR | C306 | 20.85 | 2254.851 | 13.3 | Q13490\|BIRC2_HUMAN |
| NKHEMVVYEAASAIVNLPGC*SAK | C280 | 23.68 | 2757.308 | 29.5 | Q9Y678\|COPG1_HUMAN |
| NYMSNPSYNYEIVNRASLAC*GPM(+15.99)VK | C3389 | 22.35 | 3163.402 | 28.9 | Q14204\|DYHC1_HUMAN |
| QLAEPQC*SFEDLNNEK | C447 | 20.89 | 2190.935 | 21.5 | P0CG33\|GOG6D_HUMAN:A6NDN3\|GOG6B_HUMAN:Q9NYA3\|GOG6A_HUMAN:A6NDK9\|GOG6C_HUMAN |
| RSNSC*SSISVASC(+57.02)ISEWEQK | C717 | 20.09 | 2584.115 | 13.4 | Q02241\|KIF23_HUMAN |
| SHFEQWGTLTDC*VVM(+15.99)R | C43 | 22.68 | 2250.965 | 28.6 | P09651\|ROA1_HUMAN:Q32P51\|RA1L2_HUMAN |
| SLAAYC*VIC(+57.02)C(+57.02)RR | C10 | 22.75 | 1797.794 | 18.2 | Q96JX3\|SRAC1_HUMAN |
| SLSGSSPC*PK | C785 | 30.87 | 1288.558 | 5.1 | Q9UQ35\|SRRM2_HUMAN |
| SPHTC*WQVFVTSSGK | C351 | 33.06 | 1989.887 | 20.5 | Q5JSJ4\|INT6L_HUMAN |
| SQAGVSSGAPPGRNSFYMGTC*QDEPEQLDDWNRIAELQQR | C1907 | 24.35 | 4764.118 | 35.1 | Q14980\|NUMA1_HUMAN |
| SSGRC(+57.02)WIFSC*LNVM(+15.99)R | C78 | 23.16 | 2157.937 | 23.8 | Q13867\|BLMH_HUMAN |
| SVMDATQIAGLNC*LRLM(+15.99)NETTAVALAYGIYK | C167 | 22.9 | 3672.781 | 23.3 | P34932\|HSP74_HUMAN |
| TELATYPGIIVSNIC*PGPVQSNIVENSLAGEVTKTIGNNGDQSHK | C233 | 25.96 | 4991.443 | 38.2 | Q9Y394\|DHRS7_HUMAN |
| TSGVVTSC*TGVLPQLSMVK | C315 | 20.02 | 2233.095 | 34.9 | P49736\|MCM2_HUMAN |
| VC*FQIELAHWFYLDFYMQNTPGLPQC(+57.02)GIR | C36 | 28.44 | 3872.776 | 40.7 | Q8IU60\|DCP2_HUMAN |
| VLGC*PEALTGSYKSM(+15.99)FQK | C198 | 24.61 | 2301.063 | 18.9 | Q14181\|DPOA2_HUMAN |
| VQSFQMKRC*LDK | C33 | 20.52 | 1808.852 | 22.7 | Q96QK1\|VPS35_HUMAN |
| YKPESEELTAERITEFC*HRFLEGK | C343 | 22.39 | 3238.522 | 22.9 | P07237\|PDIA1_HUMAN |
| YQHISFMPTLHC*VMHNGAQK | C724 | 20.54 | 2668.196 | 26.9 | Q9C0B9\|ZCHC2_HUMAN |
